# Design of Block-Scrambling-Based privacy protection mechanism in healthcare using fusion of transfer learning models with Hippopotamus optimization algorithm

**DOI:** 10.1038/s41598-025-04931-3

**Published:** 2025-07-01

**Authors:** Ghada Moh. Samir Elhessewi, Mohammed Yahya Alzahrani, Mohammad Alamgeer, Abdulbasit A. Darem, Da’ad Albalawneh, Mohammed Alqahtani, Mutasim Al Sadig, Sultan Alanazi

**Affiliations:** 1 Department of Health Sciences, College of Health and Rehabilitation Sciences, Princess Nourah bint Abdulrahman University, P.O. Box 84428, Riyadh, 11671 Saudi Arabia; 2 Faculty of Computing and Information, Al-Baha University, Al-Baha, Saudi Arabia; 3 Department of Information Systems, Applied College at Mahayil, King Khalid University, Abha, Saudi Arabia; 4 Center for Scientific Research and Entrepreneurship, Northern Border University, Arar, 73213 Saudi Arabia; 5 Department of Computer Science, University College in Umluj, University of Tabuk, Tabuk, Saudi Arabia; 6 Department of Information System and Cyber Security, College of Computing and Information Technology, University of Bisha, Bisha, 61922 Saudi Arabia; 7 Department of Computer Science, College of Science, Majmaah University, Al Majmaah, 11952 Saudi Arabia; 8 Department of Computer Science, College of Computer Engineering and Sciences, Prince Sattam bin Abdulaziz University, Al-Kharj, 16273 Saudi Arabia

**Keywords:** Skin lesion classification, Block-Scrambling-Based encryption, Fusion of transfer learning, Hippopotamus optimization, Computer science, Information technology

## Abstract

In the human body, the skin is the main organ. Nearly 30–70% of individuals globally have skin-related health issues, for whom efficient and effective analysis is essential. A general method dermatologists use for analyzing skin illnesses is dermoscopy, which permits surveillance of the hidden structures of skin injuries, i.e., an area suffering from an illness whose effects are unseen to the naked eye. Dermoscopy is generally employed for cancers and other kinds of skin cancers with pigment. Yet, access to a dermoscopy is demanding in resource-poor areas and unnecessary for many general skin diseases. So, developing an effective skin disease analysis method that depends upon effortlessly accessible clinical imaging would be helpful and deliver lower-cost, common access to many individuals. Recently, computer-aided diagnosis (CAD) approaches have been effectively employed to detect skin cancers in dermatoscopic imaging. The CAD-based techniques will be beneficial for helping professionals detect and classify skin lesions. This paper presents an Advanced Skin Lesion Classification using Block-Scrambling-Based Encryption with a Fusion of Transfer Learning Models and a Hippopotamus Optimization (SLCBSBE-FTLHO) model. The main aim of the SLCBSBE-FTLHO model relies on automating the diagnostic procedures of skin lesions using optimal DL approaches. At first, the block-scrambling-based encryption (BSBE) technique is utilized in the image encryption pre-processing stage, and then the decryption process is performed. The feature extraction process employs the fusion of MobileNetV2, GoogLeNet, and AlexNet techniques. Furthermore, the conditional variational autoencoder (CVAE) method is implemented for skin lesion classification. To optimize the CVAE model performance, the hippopotamus optimization (HO) model is utilized for hyperparameter tuning to ensure that the optimum hyperparameters are chosen for enhanced accuracy. To exhibit the improved performance of the SLCBSBE-FTLHO approach, a comprehensive experimental analysis is conducted under the skin cancer ISIC dataset. The comparative study of the SLCBSBE-FTLHO approach portrayed a superior accuracy value of 99.48% over existing models.

## Introduction

Generally, the skin is the largest organ that covers bones, muscles, and all over the body. The function of the skin in the human body has greater significance because minor changes in its functioning may affect other body parts^1^. Skin is uncovered, which is why many infections and skin diseases occur. Therefore, skin diseases need the utmost attention. The infected area on the skin is referred to as a lesion^2^. The skin is exposed directly to the atmosphere, which leads to more skin diseases. It infuses all cultures, arises at all ages, and affects the health of over 30–70% of people^3^. The epidermis has melanocytes, which produce melanin at a very unusual rate under any circumstance. A widespread method dermatologists utilize for identifying skin infections is dermoscopy, which allows observation of the hidden structures of skin lesions, for example, an area that suffers from disease, whose effects are unseen to the naked eye^4^. Dermoscopy is typically employed for melanomas and other types of skin cancers with pigment. However, accessing a dermoscopy in needy places is hard and pointless for more common skin diseases^5^. Hence, advancing the efficient skin disease diagnosis method based on easily retrieved medical images is beneficial and provides cheap and worldwide access to many individuals. Dermoscopy uses polarised light that reduces the stratum corneum’s translucency^6^. Appropriate analysis of these dermoscopic images leads to improved medical analytic precision. Initial analysis of skin lesions is crucial for improved treatment^7^. Currently, the medical sector relies more on computer-aided diagnosis (CAD). The initial recognition of skin disease is more difficult for inexperienced dermatologists. Integrating digital image processing for skin cancer recognition can diagnose without physical contact with the skin^8^. Hence, advancing the CAD System has become an important research area in the medical field. An early study on the CAD methods for skin lesions generally connects with pigmented skin disease (e.g., melanoma) classification and detection, whose predicted or detected results did not accurately advance the doctors’ diagnosis. With the improvement of CV and AI, many investigations use computers to analyze anatomical data^9^. DL and ML are crucial in the medical domain for automating many procedures. It revealed that dermoscopy may worsen the diagnostic precision for inexperienced dermatologists. The growth of automatic diagnostic systems for skin lesion screening^10^. It provides promising reproducibility of analytical outcomes and increases the speed of the diagnosis procedure. Moreover, automated diagnosis applications have aided in reducing first-time diagnostic faults.

This paper presents an Advanced Skin Lesion Classification using Block-Scrambling-Based Encryption with a Fusion of Transfer Learning Models and a Hippopotamus Optimization (SLCBSBE-FTLHO) model. The main aim of the SLCBSBE-FTLHO model relies on automating the diagnostic procedures of skin lesions using optimal DL approaches. At first, the block-scrambling-based encryption (BSBE) technique is utilized in the image encryption pre-processing stage, and then the decryption process is performed. The feature extraction process employs the fusion of MobileNetV2, GoogLeNet, and AlexNet techniques. Furthermore, the conditional variational autoencoder (CVAE) method is implemented for skin lesion classification. To optimize the CVAE model performance, the hippopotamus optimization (HO) model is utilized for hyperparameter tuning to ensure that the optimum hyperparameters are chosen for enhanced accuracy. To exhibit the improved performance of the SLCBSBE-FTLHO approach, a comprehensive experimental analysis is conducted under the skin cancer ISIC dataset.

- The SLCBSBE-FTLHO model utilizes the BSBE method for pre-processing to secure medical image data and ensure confidentiality. This approach improves the protection of sensitive medical images during transmission and storage. By integrating BSBE, the model strengthens privacy while maintaining the integrity of the data for additional analysis.
- The SLCBSBE-FTLHO approach integrates MobileNetV2, GoogLeNet, and AlexNet to extract features from skin lesion images effectively, enhancing accuracy and efficiency. This fusion model utilizes the merits of each network to capture diverse and relevant features. It improves the overall robustness of the model in detecting key patterns in medical image analysis.
- The SLCBSBE-FTLHO method employs the CVAE technique for precise skin lesion classification, enabling accurate differentiation between diverse lesion types. The CVAE improves classification performance under varying conditions by conditioning the extracted features. This approach confirms reliable and efficient skin lesion diagnosis.
- The SLCBSBE-FTLHO methodology implements the HO technique to fine-tune model parameters, enhancing the overall classification accuracy. Using this optimization technique, the model effectively explores the parameter space for optimal performance. HO improves the robustness and precision of the classification task.
- The SLCBSBE-FTLHO model integrates BSBE for secure data encryption, incorporating advanced DL methods such as MobileNetV2, GoogLeNet, and AlexNet for effective feature extraction. The model utilizes CVAE for accurate skin lesion classification while presenting a novel HO-based tuning method to optimize model parameters. This novel integration ensures improved security and performance, addressing critical challenges in medical image analysis.

## Review of literature

In^11^, a BC-based method is planned to classify skin diseases aided by an enhanced DL technique. The skin disease is identified depending on the skin images kept in the BC in a dataset. Additionally, a fresh segmentation method was established to excerpt the lesions from the surroundings of the skin. DeepJoint segmentation, adapted by the presence of the Kumar-Hassebrooks distance, is used for segmentation. Afterwards, feature extraction and data augmentation were implemented, and the attributes defined were exposed to the LeNet for identification. Pingulkar et al.^12^ proposed an automatic classification method to detect skin disease that uses DL procedures to diagnose skin lesions precisely. This method enables the sharing of secure diagnostic data between clinical experts, supporting the cooperative planning of the treatment. Identifying the profound significance of confidentiality and data security in the medical field, the classification uses advanced security measures for protecting the patient’s sensitive information while transferring medical data. Kolluri et al.^13^ aimed to transform the estimation of skin diseases in patients. The Convolutional Neural Network method and Exception models are combined through transfer learning (TL) to provide a sturdy estimation structure. The TL model utilizes pre-trained techniques on patient-specific information to stimulate training and improve accuracy. This study attains a new tactic to address data security problems by using BC technology to secure patient data by ensuring integrity and transparency. Rajeshkumar et al.^14^ proposed a BC-aided Homomorphic Encryption Method for Skin Lesion Diagnosis employing an Optimum DL (BHESKD-ODL) technique. The proposed BHESKD-ODL method accomplishes security and appropriate identification of skin lesion images, utilizing BC to store the patient’s medical image to limit accessibility to intruders or third-party users. Furthermore, the BHESKD-ODL model safeguards medical images through the Mayfly Optimization (MFO) procedure by the Homomorphic Encryption (HE) method. Thwin and Park^15^ introduced a skin lesion detection model, which connects the abilities of 3 progressed DL methods: Inception-V3, ResNet-50, and VGG16. Incorporating these techniques within the ensemble, this method optimizes their strengths for improved identification precision and sturdiness. Each method in the ensemble makes its characteristic contributions and has experienced pretraining on ImageNet and consequent fine-tuning through dermoscopic images. Islam et al.^16^ presented a sophisticated pipeline for building an excellent healthcare system by combining security. DL was introduced recently in skin lesion classification. There are several mechanisms to build CAD for skin lesions. A CAD system detects skin cancer with the help of a smartphone camera. The authors^17^ investigated a convolutional neural network method to categorize skin illnesses depending on the fusion model. The fusion model and deep feature fusion reinforce the capability of the feature extraction model.

Khan et al.^18^ proposed a new DL procedure for detecting skin cancer. The CAD skin system categorizes and detects skin lesions through AEs and DCNN. The CAD was developed and designed using the current pre-processing method. It is a fusion of gamma correction, multiscale retinex, contrast-limited adaptive histogram equalization, and sharp masking. They executed data augmentation strategies to deal with the unstable dataset. Moreover, a Quantum SVM (QSVM) procedure is incorporated into the last detection step. Dharani Devi and Jeyalakshmi^19^ present a TL–based FL approach utilizing ResNet and domain adversarial training across decentralized medical centres while preserving data privacy and addressing domain shift challenges. Amaizu et al.^20^ propose FedViTBloc. This secure framework incorporates FL, ViT, and blockchain (BC) to enable decentralized medical image analysis while conserving patient privacy through homomorphic encryption and differential privacy. Bárcena et al.^21^ present an FL approach by utilizing a fuzzy regression tree (FRT) model to improve model interpretability and privacy, and compare its performance with a multilayer perceptron neural network (MLP-NN), illustrating competitive RMSE scores and improved generalization in distributed settings. Yao et al.^22^ proposed the federated learning (FL) with a shuffle model and differential privacy in edge computing environments (FedShufde) framework. Gamiz et al.^23^ presented a privacy-preserving and robust FL (PRoT-FL) framework that coordinates multiple training sessions using a secure protocol integrated with BC for auditability, ensuring protection against privacy attacks and maintaining model accuracy. Bezanjani, Ghafouri, and Gholamrezaei^24^ introduce a three-phase method integrating BC-based encryption for secure data transactions, request pattern recognition for detecting unauthorized access, and bidirectional long short-term memory (BiLSTM)-based feature selection. Xu et al.^25^ propose AAQ-PEKS, a lattice-based, quantum-resistant searchable encryption scheme with attribute-based access control for secure EMR sharing in IoMT, ensuring IND-CPA and IND-CKA security with superior efficiency. Khan et al.^26^ present Fed-Inforce-Fusion, a privacy-preserving FL-based intrusion detection system for IoMT networks, incorporating reinforcement learning (RL) to capture medical data patterns and a dynamic client fusion strategy. Shukla et al.^27^ present a privacy-preserving breast cancer detection method utilizing FL with a differential privacy model. Abidi, Alkhalefah, and Aboudaif^28^ propose a crossover-based multilayer perceptron (CMLP) model to detect adversarial attacks on medical records. Haripriya, Khare, and Pandey^29^ introduce a privacy-preserving medical image classification framework integrating TL and FL, introducing an adaptive aggregation method to improve model accuracy, scalability, and convergence across diverse medical datasets. Huang et al.^30^ present a personalized federated TL (PFTL) framework that integrates pre-trained global models with client-specific fine-tuning and dynamic aggregation, utilizing attention mechanisms for customized health prognosis across heterogeneous clients.

Despite crucial BC, FL, and DL improvements for skin disease detection and medical data privacy, interoperability, model generalizability, and real-time scalability exist. Many works depend on single or limited datasets, mitigating robustness across diverse domains. Privacy techniques, namely HE and DP, are integrated but often lack alignment with explainability and interpretability requirements. Furthermore, ensemble or TL models may suffer high computational costs, restricting deployment in low-resource environments. A research gap remains in developing lightweight, interpretable, and privacy-preserving frameworks that balance security, accuracy, and real-time performance in dispersed healthcare systems.

## Proposed methodology

**Fig. 1:**
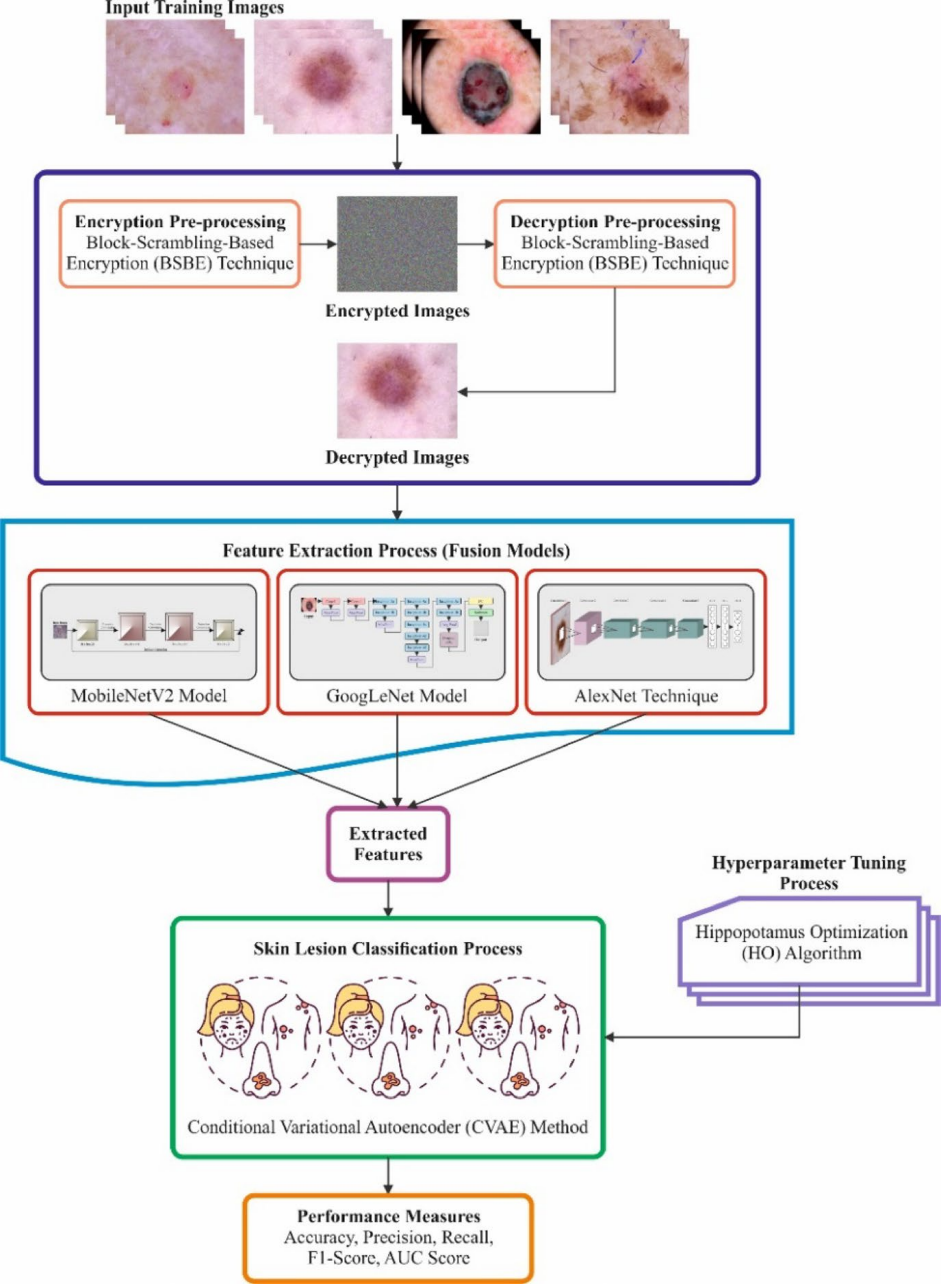
Overall working flow of the SLCBSBE-FTLHO model.

This paper presents an SLCBSBE-FTLHO approach. The approach’s primary goal is to automate the diagnostic procedures of skin lesions utilizing optimal DL models. The proposed SLCBSBE-FTLHO model involves various stages: image pre-processing, feature extraction, classification, and parameter tuning. Figure 1 depicts the overall working flow process of the SLCBSBE-FTLHO model.

### Image pre-processing

**Fig. 2:**
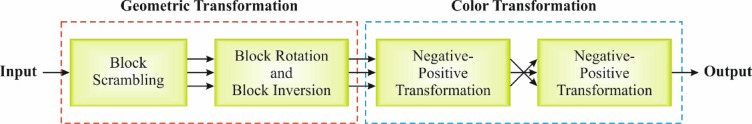
Framework of BSBE method.

At first, the BSBE technique is utilized in the image encryption pre-processing stage, and then the decryption process occurs^31^. The model is chosen for its robust mechanism for scrambling image data, which adds an extra layer of protection against potential data breaches or unauthorized access. The technique also exhibits robust safety capabilities, ensuring that medical image data remains encrypted during transmission and storage. This technique confirms that sensitive patient data remains secure without compromising the image quality for additional processing. Moreover, BSBE operates efficiently, mitigating the computational overhead usually associated with other encryption methods. The capacity to maintain data confidentiality while conserving crucial image features for analysis makes BSBE an ideal choice for medical applications, specifically in scenarios where privacy is paramount. This makes it highly appropriate for integration into sensitive domains such as medical image classification, where security and performance must be balanced. Figure 2 illustrates the BSBE framework.

The BSBE model is applied to ensure the image is encrypted safely. Using this BSBE method, a consumer should strongly transfer image $\:I$ to observers by applying virtual networking providers. However, suppose the customer fails to give the private key $\:K$ to the virtual networking providers. In that case, the confidentiality of the image, which was distributed to users, is safe, while the virtual networking providers recompress the image $\:I$. This approach mainly divided an image with $\:X$x$\:Y$ pixels into non-overlapped blocks using $\:{B}_{x}\text{x}{B}_{y}$. Then, four block‐scrambling‐based handling phases were implemented to separate the images. The method to implement the encryption image was given as shown:

Step1: Separate the image into $\:X\text{x}Y$ pixels as blocks and all blocks by $\:{B}_{x}\text{x}{B}_{y}$ pixels. Furthermore, the divided block is changed randomly using a random integer produced by the private key $\:{K}_{1},L$ bit per pixel. $\:{K}_{1}$ has commonly been used for all colour components.

Step2: Invert and rotate each block randomly using a random integer produced by the key $\:{K}_{2}$, which has typically been applied for all colour elements.

Step3: Implement negative-positive change for each of the blocks by $\:{K}_{3}$. However, $\:{K}_{3}$ is usually used for all colour components. The converted value of a pixel from the $\:{i}^{th}$ block $\:{B}_{i},{p}^{{\prime\:}}$, is measured as provided under

1 $$\:{p}^{{\prime\:}}=\left\{\begin{array}{ll}p&\:\left(r\right(i)=0),\\\:p\oplus\:({2}^{L}-1)&\:\left(r\right(i)=1).\end{array}\right.$$

Now, $\:r\left(i\right)$ suggests a random binary integer generated by $\:{K}_{3}$, and $\:p\in\:{B}_{i}$ indicates the pixel value of the new image using $\:L$ bit per pixel. The occurrence probability value $\:P\left(r\left(i\right)\right)=0.5$ is applied to invert the bits randomly

Step4: Rearrange the three colour components from each block using a number randomly selected between 6 integers by key $\:{K}_{4}.$

The sample of the image encryption is $\:({B}_{x}={B}_{y}=16)$. So, it is connected to the BSBE image for the following purposes.

(i) The encrypted images are suitable for JPEG.
(ii) The compression efficiency for encrypting the images is equal to that of the novel in JPEG.
(iii) Sturdiness against some assaults is determined.

### Fusion-based feature extraction process

The feature extraction model employs the fusion of the MobileNetV2, GoogLeNet, and AlexNet techniques. MobileNetV2 is lightweight and effective, making it ideal for resource-constrained environments while maintaining high performance in feature extraction. GoogLeNet, with its Inception module, outperforms in capturing multiscale features, allowing it to process complex and varied image patterns effectively. AlexNet, though older, has proven to be highly efficient in extracting deep features from images, giving robust baseline performance. Integrating these models ensures robustness in feature extraction, as each model compensates for the limitations of the others, resulting in more accurate and comprehensive representations of skin lesion images. This fusion not only enhances classification accuracy but also improves the capability of the model to generalize across diverse datasets, making it an ideal choice for medical image analysis.

**MobileNetV2 model**

**Fig. 3:**
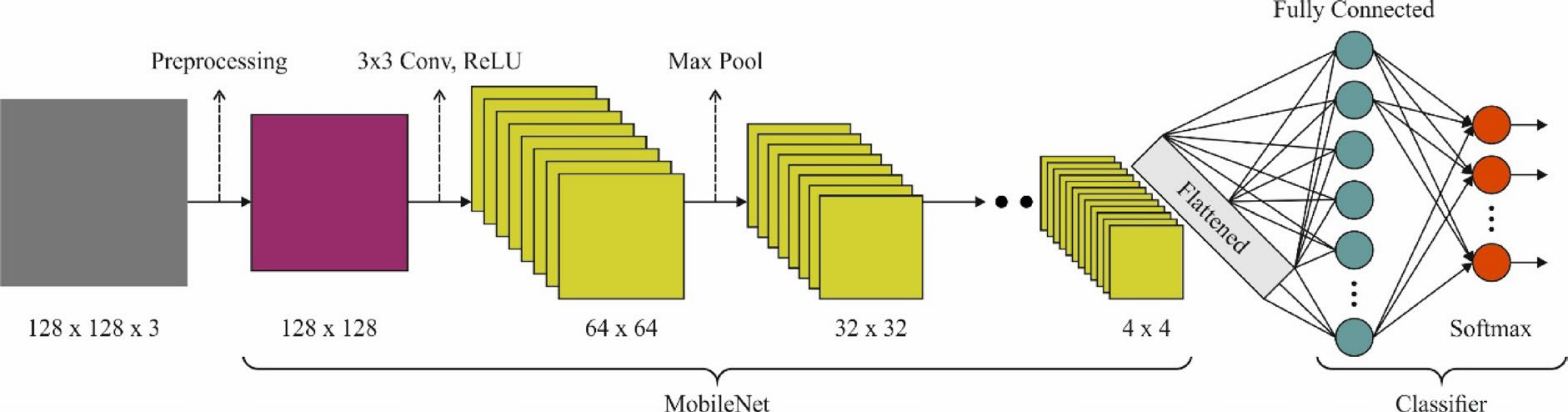
Architecture of MobileNetV2 model.

The MobileNetV2 framework is applied to images given as input. This model starts with the image phase, whereas the images with dissimilar shapes and textures are gathered as input data for the method. Then, a pre-trained MobileNetV2 method is loaded to remove the main characteristics from the input images^32^. During this training stage, the MobileNetV2 structure incorporates some major modules. Initially, feature extraction is carried out to remove essential features from the images utilizing a primary convolution with a 3×3 filter. A main module in this phase is the inverted Residual Blocks, which are convolutional blocks that use depth-wise separable convolution to decrease the parameter counts and enhance computational complexity. These blocks utilize shortcut links to preserve feature information in the propagation method. Then, the bottleneck layers act as the basis for MobileNetV2. These layers use inverted residual links, which permit information to remain unchanged efficiently regardless of the small parameter counts. These layers further improve the feature representation ability without considerably increasing computational cost. Afterwards, the features are well‐removed; the following phase is the fully connected layers in charge of the last classification. During this layer, the ReLU is applied in the feature mapping process. Figure 3 indicates the MobileNetV2 framework.

**GoogLeNet method**

**Fig. 4:**
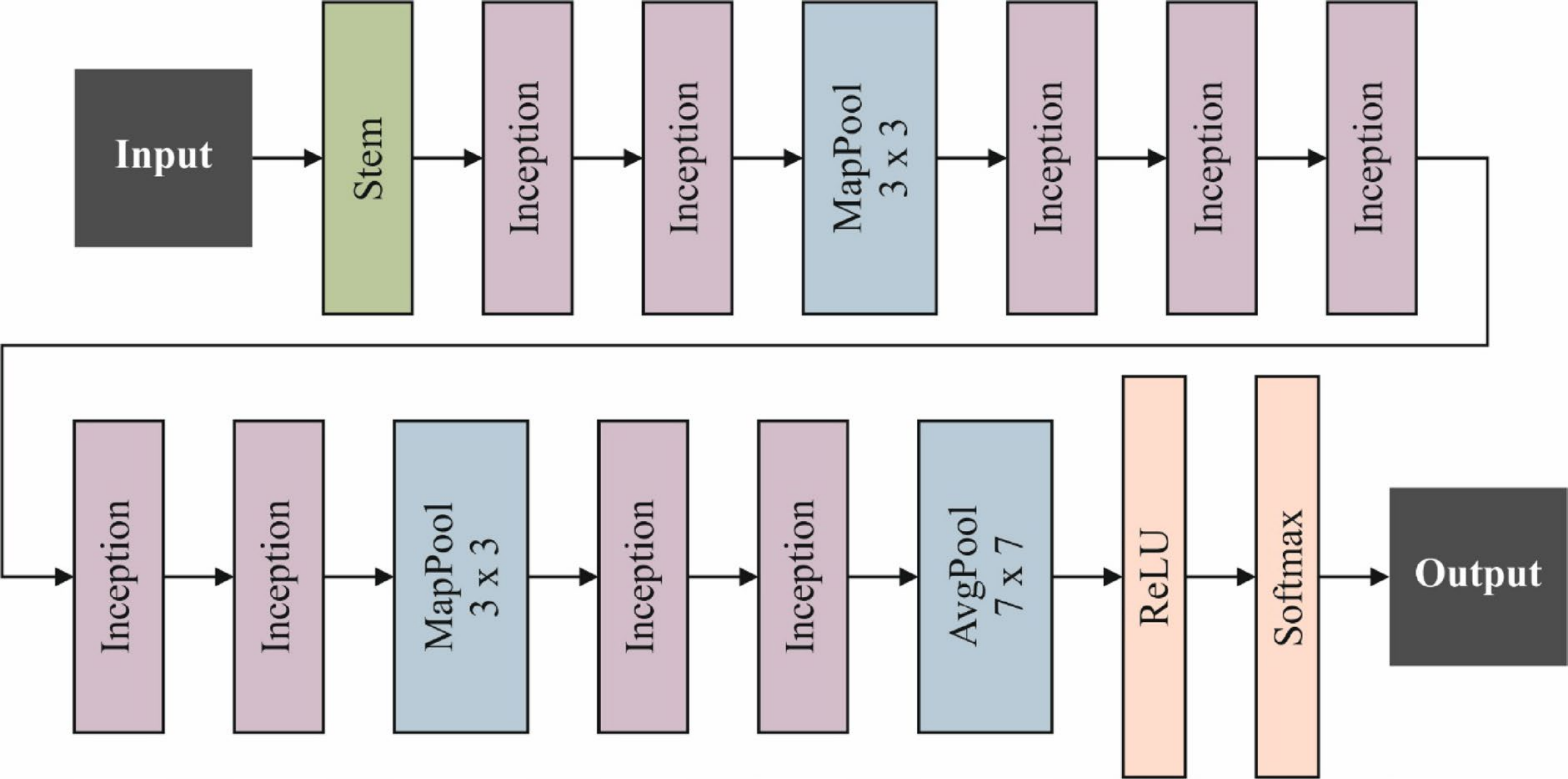
Architecture of the GoogLeNet method.

The GoogLeNet Architecture is applied to classify images into some types according to visual features removed by the method. This model starts with the image phase, where different images with dissimilar textures and shapes are gathered as input data. Then, a pre-trained GoogLeNet method is loaded, acting as a feature extractor in the training stage. According to CNN, GoogLeNet is a well-known DL method for its Inception structure, which aims to improve computational complexity while preserving higher image precision. During this training stage, GoogLeNet contains key layers important in feature extraction. The model begins with convolutional layers that use many receptive fields to incorporate 1×1, 3×3, and 5×5 convolutions inside inception elements. This allows the model to capture feature information at different scales without considerably increasing the parameter counts. Furthermore, the ReLU is applied to present non‐linearity in the network and enhance feature representation abilities. Then, the removed features are handled through inception Components, which create the basis of GoogLeNet. These components use parallel convolutional filters to capture textural and spatial features at various detail levels in the input image. This incorporation gives higher flexibility in processing objects with composite texture and shape variations. After that, the pooling procedure is performed, whereas numerous pooling layers with max-pooling are applied to reduce feature sizes without losing significant information. This pooling targets to enhance computational cost and decrease the probability of overfitting. Moreover, a dropout layer is added to alleviate overfitting in training, thus improving the generalization method to novel data. After passing through some feature extraction phases, the process utilizes the softmax activation function to make likelihoods for every class in the multi-classification phase. Figure 4 specifies the GoogLeNet model.

**AlexNet model**.

**Fig. 5:**
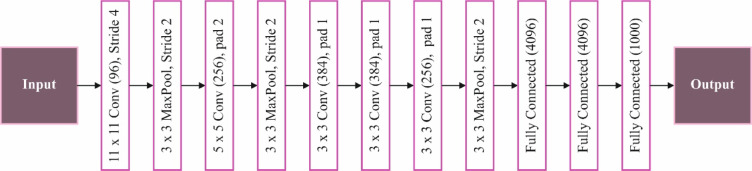
Flow of the AlexNet method.

AlexNet is a deeper CNN structure that significantly improves computer vision (CV) tasks. This architecture has eight fully connected layers and five convolutional layers. To gather lower-level characteristics, the initial convolutional layer benefits from the revolutionary receptive field for CV tasks, namely, the image category^33^. The first convolutional layer uses a wide receptive field to collect low-level features, which is revolutionary for computer vision tasks like picture categorization. The following layers use small receptive fields to capture complex and conceptual components. In contrast, the first convolutional layer applies a larger receptive field for recording lower-level features such as textures and edges. AlexNet primarily used the ReLU. Furthermore, dropout regularization is used to stop overfitting after training. The success of AlexNet on the ImageNet dataset, consisting of more than a million images, shows the ability of deeper neural networks in image classification tasks and opens the vision field. Figure 5 portrays the flow of the AlexNet technique.

### Classification model using CVAE technique

**Fig. 6:**
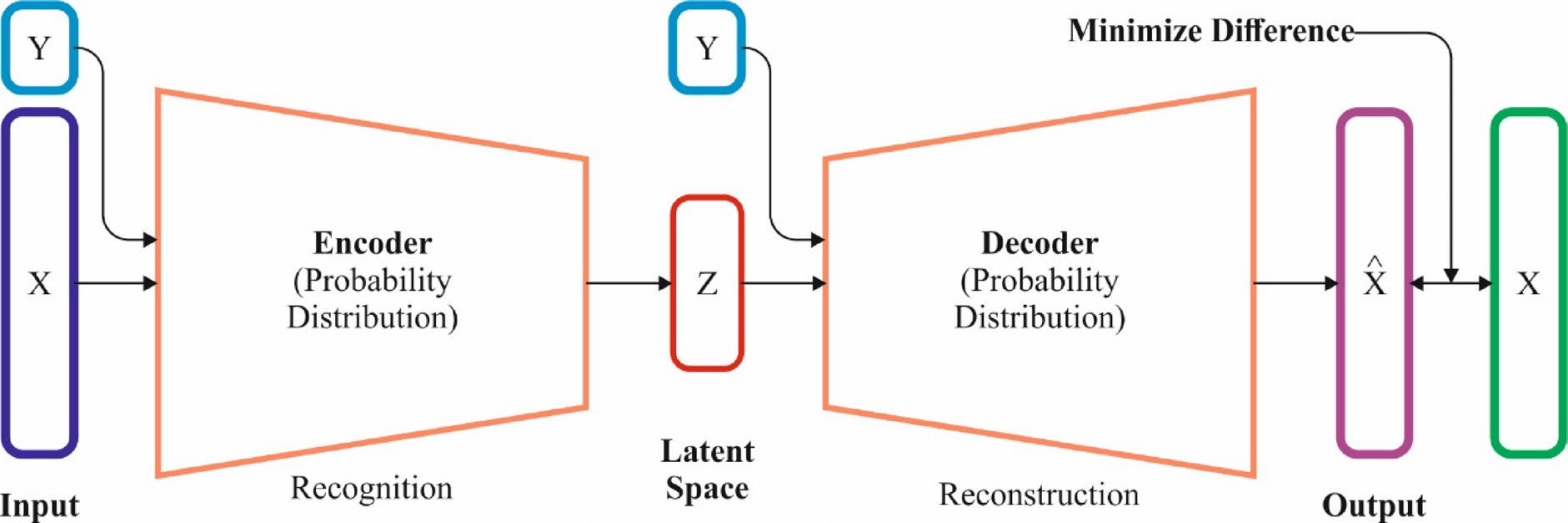
Structure of the CVAE model.

In this section, the SLCBSBE-FTLHO model employs the CVAE method for the skin lesion classification^34^. This model is chosen because it can generate highly accurate, probabilistic, and interpretable outputs. Unlike conventional classifiers, this technique can model intrinsic and diverse data dispersions by learning latent variables that capture the underlying features of skin lesions. This enables the model to classify images more robustly, without noise or limited data. The conditional aspect of CVAE allows it to condition the output based on specific attributes (e.g., lesion characteristics), making it highly appropriate for medical image classification where context and condition are crucial. Furthermore, CVAE’s generative nature improves its capability to handle data variability and enhance classification performance over standard discriminative models. This flexibility and capacity to generate realistic samples make CVAE appropriate for skin lesion classification tasks. Figure 6 signifies the structure of the CVAE model.

AE is a neural network that reconstructs and compresses original data. An AE contains a decoder $\:{p}_{\theta\:}\left(x|z\right)\:$and an encoder $\:q\varphi\:\left(z|x\right)$. The encoder compresses the input $\:x$ into latent variables $\:z$, and the decoder rebuilds the input $\:x$ from $\:z$. The AE aims to learn parameters $\:\theta\:$ and $\:\varphi\:$ to reduce the dissimilarity between the input and the recreated output. Meanwhile, $\:z$ contains a smaller dimension than $\:x$, so it cannot easily output the input copy. Then, the encoding is learned to function as the feature extractor and the decoder as the generator. AE’s variants consist of VAE and CVAE.

VAE also learns the dispersal of latent variables $\:{p}_{\theta\:}\left(z\right|x)$ from the input $\:x$; however, in VAE, this distribution is demonstrated as a Gaussian distribution. The encoding outputs the variance vector $\:{\sigma\:}^{2},\:$the mean vector $\:\mu\:$, and the latent variables $\:z$ are tested depending on these, as presented as:

2 $$\:z\sim\:\mathcal{N}\left(\mu\:\left(x\right),\:{\sigma\:}^{2}\left(x\right)\right)$$

The decoder then recreates the latent variables $\:z$ to the original sizes. The VAE’s loss function is stated as:

3 $$\:\mathcal{L}(\theta \:,\:\varphi \: x)=-{\mathbb{E}}_{q \varphi \:(z|x)} [\text{log} \:p(Xz)]+{D}_{KL} (q\varphi \:(z|x)||p(z))$$

4 $$\:\mathcal{L}(\theta\:,\:\varphi\: x)=-{\mathbb{E}}_{{q}_{\phi\:}(z|x)}[\text{log}\:p(Xz)]+{D}_{KL}(q\varphi\:(z|x)||p(z))$$

The amount of KL divergence $\:{D}_{KL}\left(q\left(z|x\right)p\left(z\right)\right)$ among the latent distribution $\:p\left(zx\right)$ and the previous distribution $\:p\left(z\right)$, and the reconstruction error. This guarantees that the latent variables follow a Gaussian distribution, permitting closer latent variables to make related outputs. CVAE extends VAE to make data according to particular conditions (for example, classes). The loss function for CVAE in training provided classes $\:c$ is stated as:

5 $$\:\mathcal{L}(\theta\:,\:\varphi\:;x,\:c)=-{\mathbb{E}}_{q\varphi\:(z|x,c)}[\text{log}{p}_{\theta\:}(x|z,\:c)]+{D}_{KL}(q\varphi\:(z|x,\:c)||p(z))$$

Unlike VAE networks, CVAE inputs classes into the encoder and decoder. A model for connecting class $\:c$ with input $\:x$ and the latent variables $\:z$ was presented. Including the class in the training makes producing data from the distribution according to the specified class after training promising.

### HO-based parameter tuning model

**Fig. 7:**
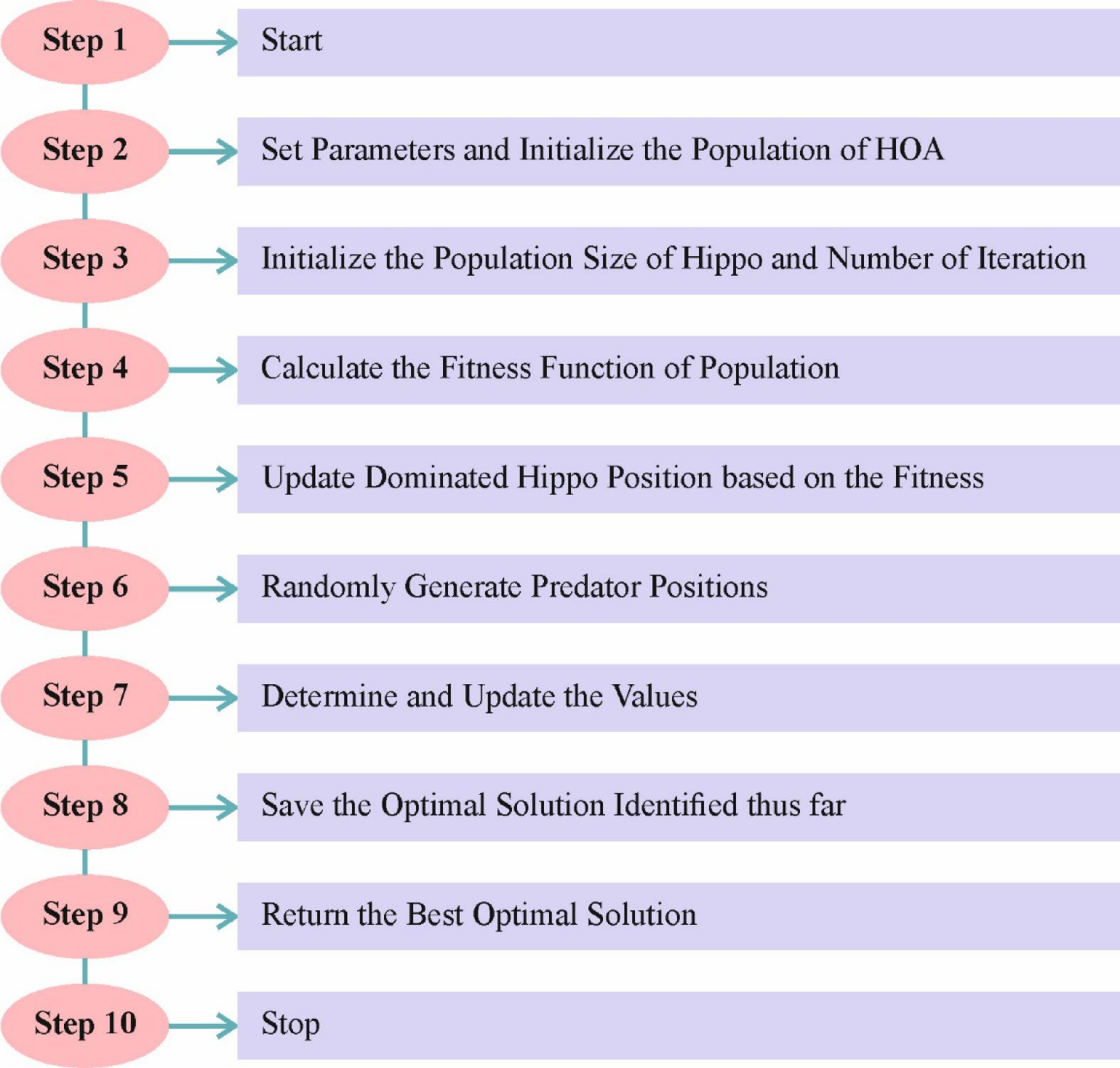
Steps involved in the HO methodology.

For optimizing the CVAE model performance, the HO approach is utilized for hyperparameter tuning to ensure that the best hyperparameters are selected for enhanced accuracy^35^. This approach is chosen due to its effectiveness in exploring massive, complex search spaces and for model parameter tuning. Unlike conventional optimization algorithms, this model replicates the adaptive behaviour of hippopotamuses in nature, allowing it to balance exploration and exploitation during the search process effectively. This results in faster convergence and better optimization outcomes, specifically for complex DL models. The robustness of the HO model in avoiding local minima and its capability to fine-tune parameters with minimal computational overhead make it highly appropriate for optimizing skin lesion classification models. HO has illustrated superior accuracy and efficiency in tasks requiring fine-tuning multiple hyperparameters compared to other techniques. Its unique behaviour ensures enhanced model performance, contributing to faster and more reliable predictions. Figure 7 depicts the steps involved in the HO technique.

HO is stimulated by hippopotamuses’ social behaviour and defence procedures. Like other optimizer techniques, the Hippopotamus’s location is a candidate solution to an issue. The Hippopotamus’s initial location is random, as assumed below.

6 $$\:H{O}_{ij}={L}_{j}+rand\times\:\left({U}_{j}-{L}_{j}\right)$$

Here, $\:H{O}_{ij}$ represents the location of the Hippopotamus in the $\:{j}^{th}$ dimension. $\:{U}_{j}\:$and$\:\:{L}_{j}$ represent the upper and lower limits for dimension $\:j$, respectively. $\:rand$ is a randomly generated number between $\:0$ and 1. Equation (7) provides matrix for $\:M$ hippopotamus in dimension$\:\:N$.

7 $$\:HO={\left[\begin{array}{cccc}H{O}_{\text{0,0}}&\:H{O}_{\text{0,1}}&\:\dots\:&\:H{O}_{0,N}\\\:H{O}_{{1,0}}&\:H{O}_{\text{1,1}}&\:\dots\:&\:H{O}_{1,N}\\\:?&\:?&\:\dots\:&\:?\\\:H{O}_{M,0}&\:H{O}_{M,1}&\:\dots\:&\:H{O}_{M,N}\end{array}\right]}_{M\times\:N}$$

The Hippopotamus’s location upgrade to discover the searching space contains three stages. Stage 1 displays the exploration utilizing the Hippopotamus’s social behaviour. The mathematical formulation is shown in Eq. (8).

8 $$\:HO=H{O}^{f}\cup\:H{O}^{c}\cup\:H{O}^{m}\cup\:H{O}^{L}$$

Meanwhile, $\:H{O}^{c},\:H{O}^{f},\:H{O}^{L}$, and $\:H{O}^{m}$ correspondingly embody the calves, females, leaders, and male hippopotamuses. Every Hippopotamus is considered $\:H{O}^{\:f}or$ $\:H{O}^{c}$, $\:H{O}^{m}$, or $\:H{O}^{L}$, depending upon its fitness value. For the male Hippopotamus, the location upgrade in the water bodies is set by Eq. (9).

9 $$\:H{O}_{ij}^{m}=H{O}_{ij}+{rand}\times\:\left(H{O}^{L}-{C}_{1}H{O}_{ij}\right)|i=1,2,3,\:\dots\:,\:{[}^{M}{/}_{2}]\:{and}\:j=1, 2,3,\dots\:N$$

Here, $\:{C}_{1}$ denotes a constant number between 1 and 2. For female hippopotamuses, the location upgrade and calves, i.e., $\:H{O}^{fc}={H}^{f}\cup\:{H}^{c}$, are determined by Eq. (10).

10 $$\:H{O}_{ij}^{fc}=\left.\begin{array}{c}H{O}_{ij}+{v}_{1}\times\:(H{O}^{L}-{C}_{2}R{G}^{m})\\\:H{O}_{ij}+{v}_{2}\times\:(R{G}^{m}-H{O}^{L})\\\:{L}_{j}+{ rand }\times\:({U}_{j}-{L}_{j})\end{array}\right|\begin{array}{c}T>0.6\\\:{else if rand}>0.5\\\:{else}\end{array}$$

While $\:i=1,2,3,\dots\:,\:{[}^{M}{/}_{2}]$ and $\:j=1, 2,3,\dots\:N$. $\:{v}_{1},{v}_{2}$ are produced utilizing Eq. (11), $\:T$ is made by employing Eq. (12). $\:{C}_{2}$ denotes a constant number between 1 and 2. $\:R{G}^{m}$ indicates the mean of the chosen Hippopotamus randomly from the accessible $\:M$ hippopotamus.

11 $$\:v=\left\{\begin{array}{l}{C}_{2}\times\:\overrightarrow{{rand}}+(\sim\:{\vartheta\:}_{1})\\\:2\times\:\overrightarrow{{rand}}-1\\\:\overrightarrow{{rand}}\\\:{C}_{1}\times\:\overrightarrow{{rand}}+(\sim\:{\vartheta\:}_{2})\\\:\overrightarrow{{rand}}\end{array}\right.$$

12 $$\:T={e}^{-Cu{r}_{iter}}/{\text{Max}}_{itr}$$

$\:Cu{r}_{itr}$ and $\:{\text{Max}}_{itr}$ denote the present and maximum iterations, respectively. $\:{\vartheta\:}_{1},{\vartheta\:}_{2}$ denote randomly generated numerals between $\:0$ and 1. Equations (13) and (14) will recognize the upgraded location of the Hippopotamus.

13 $$\:H{O}_{i}=\left\{\begin{array}{c}H{O}_{i}^{m}\\\:H{O}_{i}\end{array}\right.\left|\begin{array}{c}\text{fit}\:\left(H{O}_{i}^{m}\right)<\text{fit}\left(H{O}_{i}\right)\\\:\text{else}\end{array}\right.$$

14 $$\:H{O}_{i}=\left\{\begin{array}{l}H{O}_{i}^{fc}\\\:H{O}_{i}\end{array}\right.\left|\begin{array}{c}\text{fit}\:\left(H{O}_{i}^{fc}\right)<\text{fit}\left(H{O}_{i}\right)\\\:\text{else}\end{array}\right.$$

While $\:fit\left(\right)$ denotes a fitness function. Stage 2 shows exploration and represents the protection method against hunters. The predator location is set by Eq. (15).

15 $$\:{P}_{j}={L}_{j}+{rand}\times\:({U}_{j}-{L}_{j})|j=1, 2,3,\:\dots\:N$$

Utilizing Eq. (16), the distance of a precise hippopotamus from the hunter originated.

16 $$\:\overrightarrow{\text{Dist}}=\left|{P}_{j}-H{O}_{ij}\right|$$

The Hippopotamus selects its protective activity depending on the $\:\overrightarrow{\text{Dist}}$ value. Suppose it is in the adjacent locality of predator-fit $\:\left({P}_{j}\right)<\text{fit}\left(HO\right)$. In that case, the Hippopotamus will tackle or travel near the predator, as presented in Eq. (17).

17 $$\:H{O}_{ij}^{n}=\begin{array}{c}\text{l}\text{e}\text{v}{y}^{r}\oplus\:{P}_{j}+\left(\frac{b}{\left(c-d*\text{cos}\left(2\pi\:g\right)\right)}\right).\left(\frac{1}{\text{Dist}}\right)\\\:\text{l}\text{e}\text{v}{y}^{r}\oplus\:{P}_{j}+\left(\frac{b}{\left(c-d*\text{cos}\left(2\pi\:g\right)\right)}\right)\:.\:\left(\frac{1}{2*\text{Dist}+r\alpha\:nd}\right)\end{array}\left|\begin{array}{c}\text{fit}\left({P}_{j}\right)<\text{fit}\left(H{O}_{i}\right)\\\:\text{else}\end{array}\right.$$

Here $$\:i={[}^{M}{/}_{2}]+1,\:{[}^{M}{/}_{2}]+2,\dots\:,M\:\text{ and }\:j=1, 2,3,\dots\:N.$$

If the fitness value is superior to the current fitness value, then the upgraded location of the Hippopotamus is accepted as assumed by Eq. (18).

18 $$\:H{O}_{i}=\left\{\begin{array}{c}H{O}_{i}^{n}\\\:H{O}_{i}\end{array}\right.\left|\begin{array}{c}fit\:\left(H{O}_{i}^{n}\right)<fit\left(H{O}_{i}\right)\\\:else\end{array}\right.$$

Stage 3 displays the exploitation of the predator’s escape behaviour. Hippopotamuses usually hunt for adjacent water bodies to escape from the hunter. The local lower and upper limits are obtained utilizing Eq. (19).

19 $$\:{U}_{j}^{loc\alpha\:l}{=}^{{U}_{j}}/Cu{r}_{itr}{L}_{j}^{loc\alpha\:l}{=}^{{L}_{j}}/Cu{r}_{itr}$$

The upgraded location of the Hippopotamus is set by Eq. (20).

20 $$\:H{O}_{ij}^{n}=H{O}_{ij}+rand\left({L}_{j}^{loc\alpha\:l}+\alpha\:\left({U}_{j}^{loc\alpha\:l}-{L}_{j}^{loc\alpha\:l}\right)\right)$$

Here, the $\:\alpha\:$ formulation is shown in Eq. (21).

21 $$\:\alpha\:=\left\{\begin{array}{l}2\times\:\overrightarrow{rand}-1\\\:\overrightarrow{rand}\\\:\overrightarrow{randn}\end{array}\right.$$

Meanwhile, $\:\overrightarrow{randn}$ provides a randomly generated number. If the fitness value is superior to the present, it will travel to a safer location as set by Eq. (22).

22 $$\:H{O}_{i}=\left\{\begin{array}{c}H{O}_{i}^{n}\\\:H{O}_{i}\end{array}\right.\left|\begin{array}{c}fit\:\left(H{O}_{i}^{n}\right)<fit\left(H{O}_{i}\right)\\\:else\end{array}\right.$$

Algorithm 1 specifies the HO model.

**Algorithm 1:**
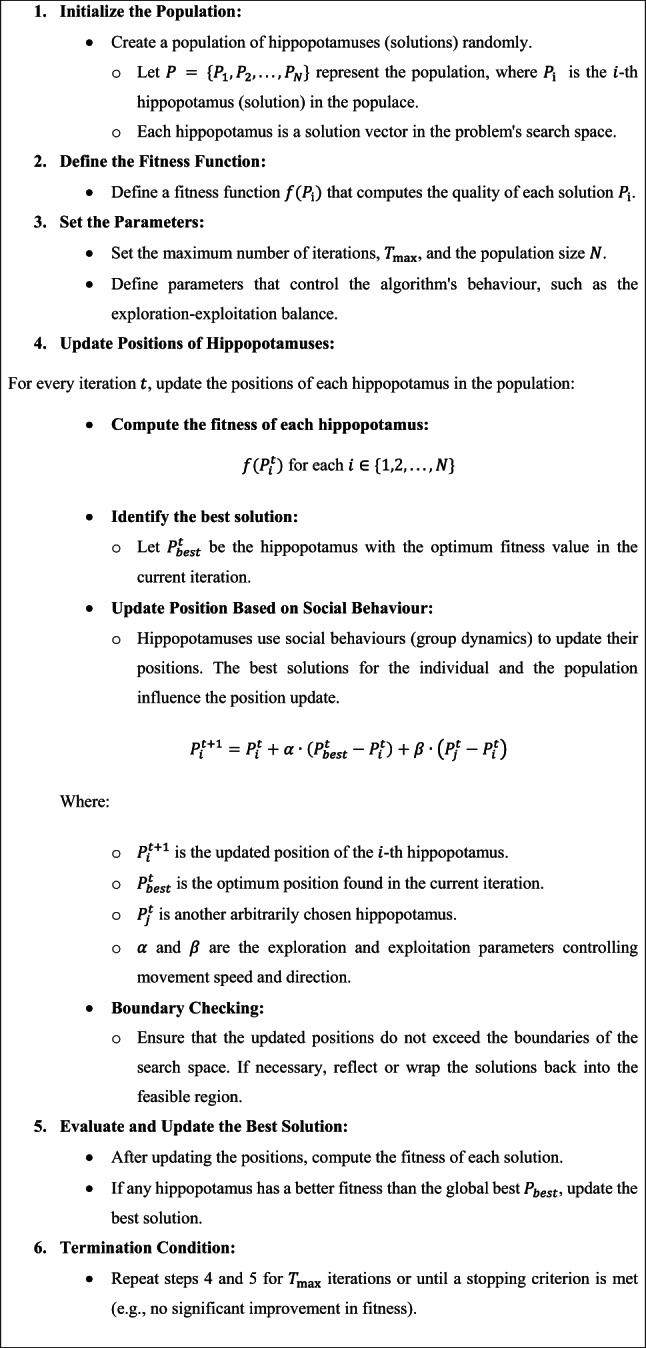
HO technique.

Fitness selection is a significant influence on manipulating the HO technique performance. The hyperparameter range process comprises the solution-encoded system to evaluate the candidate solution efficiency. The HO model studies accuracy as the primary criterion for designing the fitness function as follows:

23 $$\:Fitness\:=\:\text{m}\text{a}\text{x}\:\left(Precision\right)$$

24 $$\:Precision=\frac{TP}{TP+FP}$$

Here, $\:TP$ and $\:FP$ mean the optimistic value of true and false.

## Experimental analysis

**Fig. 8:**
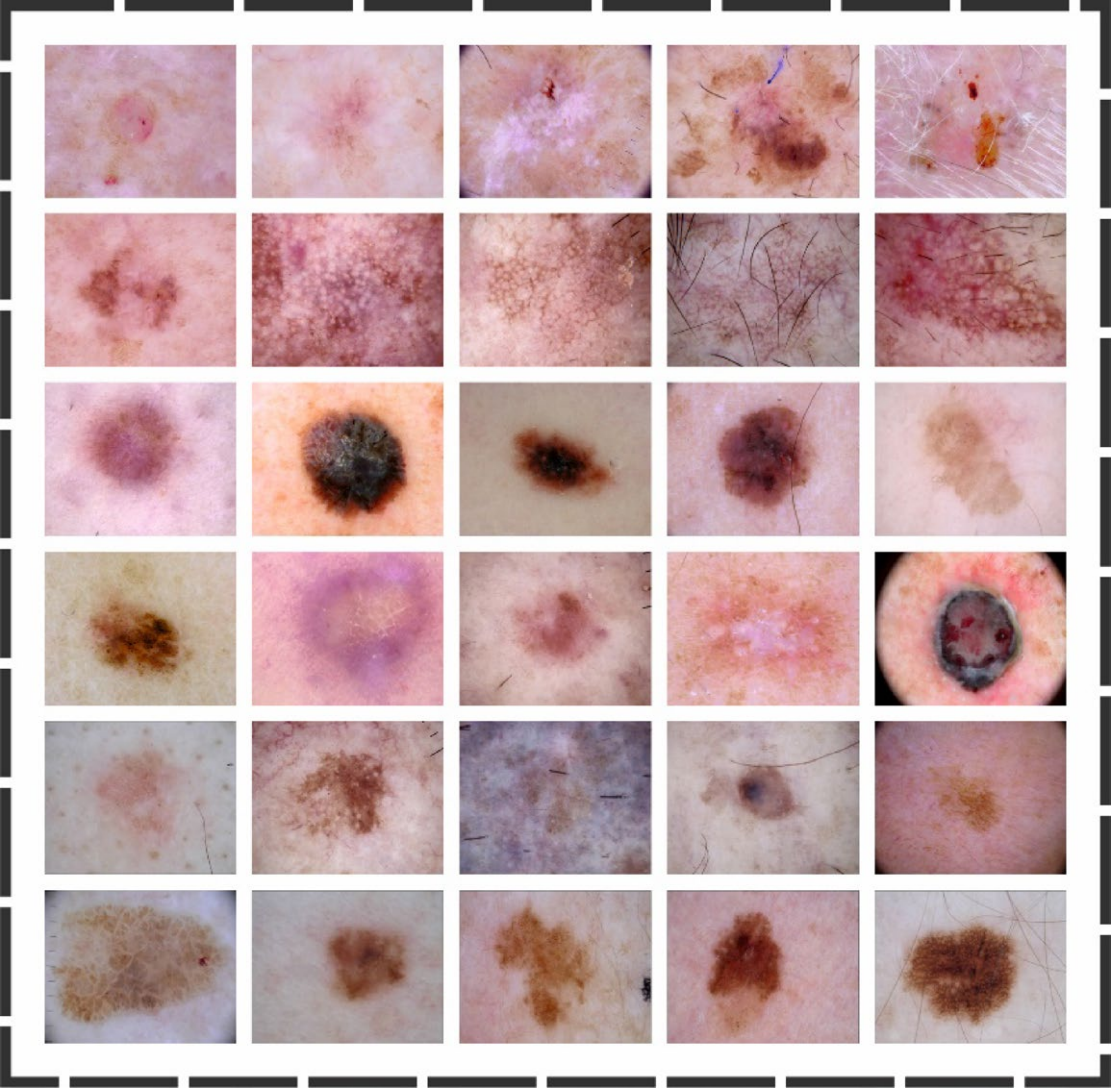
Sample images.

**Fig. 9:**
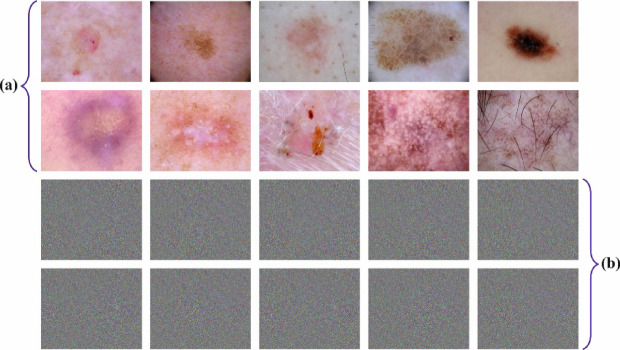
(**a**) Original images, (**b**) Encrypted images.

The experimental study of the SLCBSBE-FTLHO model is examined under the skin cancer ISIC dataset^36^. The technique is simulated using the Python 3.6.5 tool on a PC with an i5-8600k, 250GB SSD, GeForce 1050Ti 4GB, 16GB RAM, and 1 TB HDD. The parameter settings are provided: learning rate: 0.01, activation: ReLU, epoch count: 50, dropout: 0.5, and batch size: 5. This dataset holds 2239 no. of images under nine classes. The complete details of this dataset are depicted in Table 1. Figure 8 signifies the sample images. The encryption and decryption images are shown in Fig. 9.

The model’s performance is assessed using the metrics derived from the confusion matrix^37^. This matrix reveals the values for true positives (TP), true negatives (TN), false positives (FP), and false negatives (FN), which are crucial for computing key evaluation metrics such as accuracy, recall, precision, and F-measure. These metrics play a significant role in thoroughly evaluating the model. These metrics’ success rates are computed using the mathematical expressions provided in Eqs. 25–28^38^.

25 $$\:Acc{u}_{y}=\:\frac{TP+TN}{TP+TN+FP+FN}$$

26 $$\:Pre{c}_{n}=\:\frac{TP}{TP+FP}$$

27 $$\:Rec{a}_{l}=\:\frac{TP}{TP+FN}$$

28 $$\:F{1}_{Score}=\:2\times\:\frac{Pre{c}_{n}\cdot\:Rec{a}_{l}}{Pre{c}_{n}+Rec{a}_{l}}$$

**Table 1:** Details of the dataset.

Classes	Labels	No. of images
“Actinic Keratosis”	C1	114
“Basal Cell Carcinoma”	C2	376
“Dermatofibroma”	C3	95
“Melanoma”	C4	438
“Nevus”	C5	357
“Pigmented Benign “Keratosis”	C6	462
“Seborrheic Keratosis”	C7	77
“Squamous Cell Carcinoma”	C8	181
“Vascular Lesion”	C9	139
**Total Number of Images**		**2239**

**Fig. 10:**
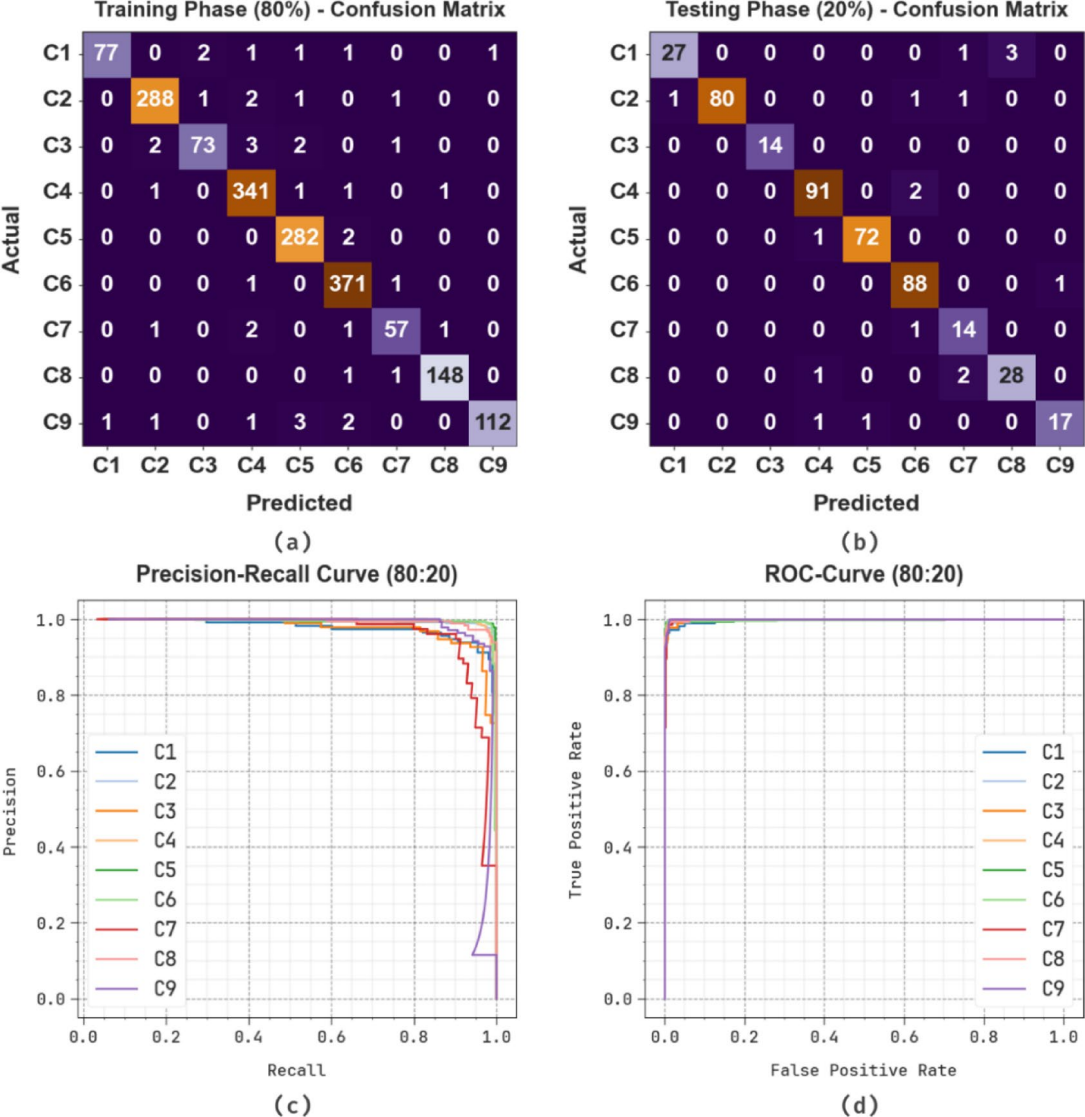
80%TRAPHA and 20%TESPHA of (**a**, **b**) confusion matrices, (**c**) curve of PR, and (**d**) curve of ROC.

Figure 10 illustrates the classification performance of the SLCBSBE-FTLHO model under 70%TRAPHA and 30%TESPHA on a multi-class dataset comprising nine classes labelled C1 to C9. In TRAPHA, the figure illustrates robust diagonal dominance, showing accurate predictions for most classes with minimal misclassifications. Likewise, the TESPHA reflects consistent performance, with high true positive values for major classes and limited off-diagonal entries, highlighting the superior generalizability of the model. Classes C4 and C5 also exhibit excellent precision and recall, with minimal confusion with other classes. Misclassifications are sparse and mostly occur between neighbouring classes with similar features. The model depicts high classification accuracy and robust generalization across all class categories.

**Fig. 11:**
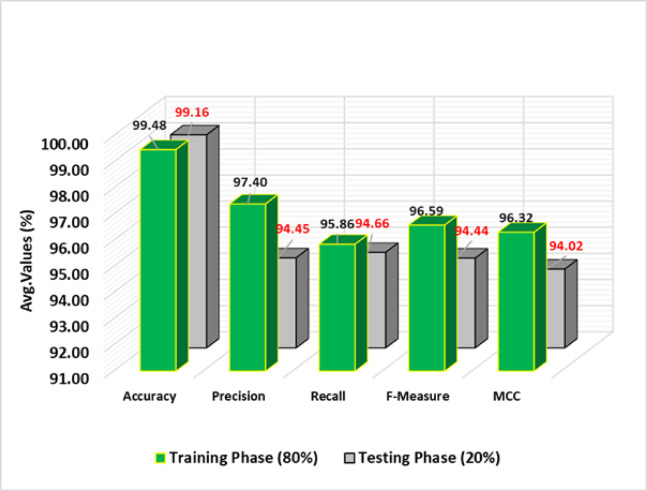
Average of SLCBSBE-FTLHO model under (**a**, **b**) 80%TRAPHA and 20%TESPHA.

Table 2; Fig. 11 represent the detection of skin cancer by the SLCBSBE-FTLHO method under 80% TRAPHA and 20% TESPHA. The result implies that the SLCBSBE-FTLHO procedure appropriately recognized the instances. By 80%TRAPHA, the SLCBSBE-FTLHO method provides an average $\:acc{u}_{y}$, $\:pre{c}_{n}$, $\:rec{a}_{l},\:{F}_{Measure}$, and $\:MCC$ of 99.48%, 97.40%, 95.86%, 96.59%, and 96.32%, correspondingly. Additionally, with 20%TESPHA, the SLCBSBE-FTLHO approach offers average $\:acc{u}_{y}$, $\:pre{c}_{n}$, $\:rec{a}_{l},\:{F}_{Measure}$, and $\:MCC$ of 99.16%, 94.45%, 94.66%, 94.44%, and 94.02%, respectively.

**Table 2:** Skin cancer detection of SLCBSBE-FTLHO model under 80%TRAPHA and 20%TESPHA.

Classes	$\:\varvec{A}\varvec{c}\varvec{c}{\varvec{u}}_{\varvec{y}}$	$\:\varvec{P}\varvec{r}\varvec{e}{\varvec{c}}_{\varvec{n}}$	$\:\varvec{R}\varvec{e}\varvec{c}{\varvec{a}}_{\varvec{l}}$	$\:{\varvec{F}}_{\varvec{m}\varvec{e}\varvec{a}\varvec{s}\varvec{u}\varvec{r}\varvec{e}}$	$\:\varvec{M}\varvec{C}\varvec{C}$
**TRAPHA (80%)**					
C1	99.61	98.72	92.77	95.65	95.50
C2	99.44	98.29	98.29	98.29	97.96
C3	99.39	96.05	90.12	92.99	92.72
C4	99.22	97.15	98.84	97.99	97.51
C5	99.44	97.24	99.30	98.26	97.93
C6	99.44	97.89	99.46	98.67	98.32
C7	99.50	93.44	91.94	92.68	92.43
C8	99.78	98.67	98.67	98.67	98.54
C9	99.50	99.12	93.33	96.14	95.92
**Average**	**99.48**	**97.40**	**95.86**	**96.59**	**96.32**
**TESPHA (20%)**					
C1	98.88	96.43	87.10	91.53	91.06
C2	99.33	100.00	96.39	98.16	97.78
C3	100.00	100.00	100.00	100.00	100.00
C4	98.88	96.81	97.85	97.33	96.62
C5	99.55	98.63	98.63	98.63	98.36
C6	98.88	95.65	98.88	97.24	96.56
C7	98.88	77.78	93.33	84.85	84.65
C8	98.66	90.32	90.32	90.32	89.60
C9	99.33	94.44	89.47	91.89	91.58
**Average**	**99.16**	**94.45**	**94.66**	**94.44**	**94.02**

**Fig. 12:**
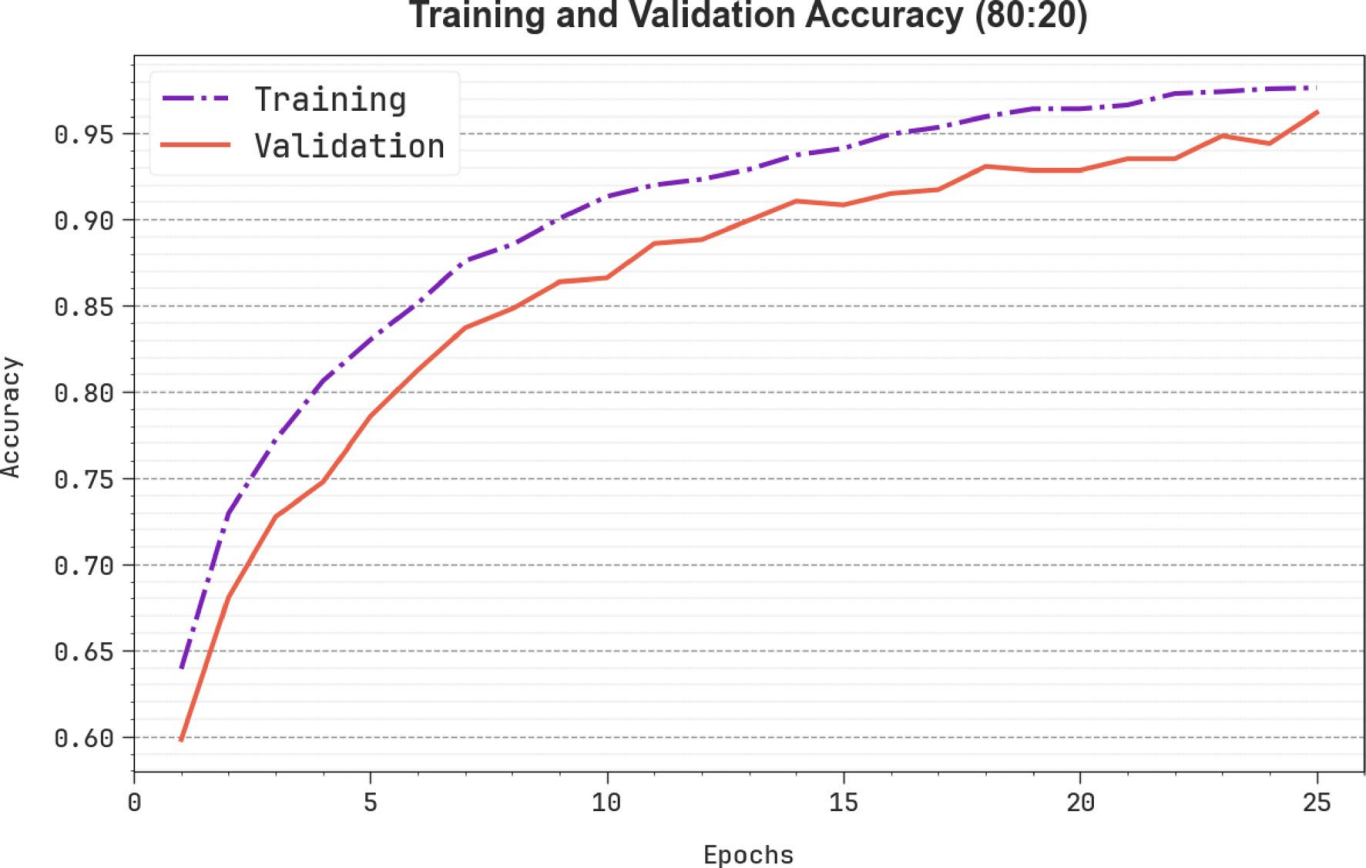
$\:Acc{u}_{y}$ curve of SLCBSBE-FTLHO method under 80:20

Figure 12 demonstrates the training (TRAN) $\:acc{u}_{y}$ and validation (VALN) $\:acc{u}_{y}$ findings of the SLCBSBE-FTLHO method under 80:20. The $\:acc{u}_{y}\:$values are intended through the 0–25 epochs interval. The figure highlighted that both the $\:acc{u}_{y}$ values exhibit an increasing propensity that informed the capability of the SLCBSBE-FTLHO method with enhanced performance through numerous iterations. Additionally, both $\:acc{u}_{y}$ remain near over the epochs, which suggests marginal overfitting and demonstrates improved performance of the SLCBSBE-FTLHO method, ensuring reliable estimation on invisible samples.

**Fig. 13:**
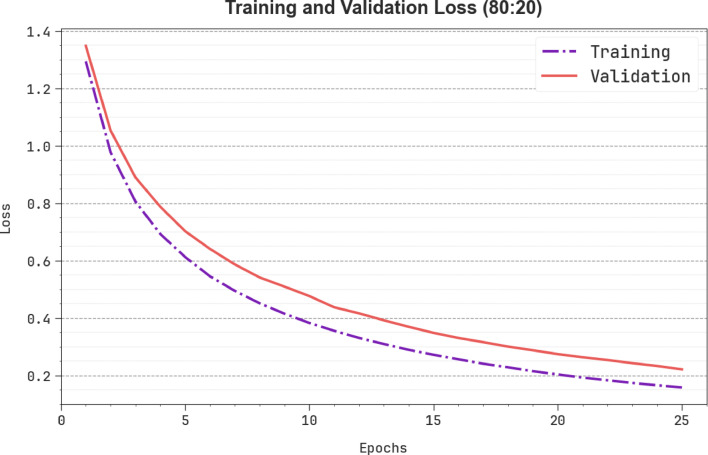
Loss curve of SLCBSBE-FTLHO method under 80:20.

In Fig. 13, the TRAN loss (TRANLOS) and VAL loss (VALNLOS) graph of the SLCBSBE-FTLHO design under 80:20 is exhibited. The loss values are figured for 0–25 epochs. Both values exemplify a reducing propensity, notifying the ability of the SLCBSBE-FTLHO method to balance a trade-off. The constant reduction in loss values assures the enhanced performance of the SLCBSBE-FTLHO method.

**Fig. 14:**
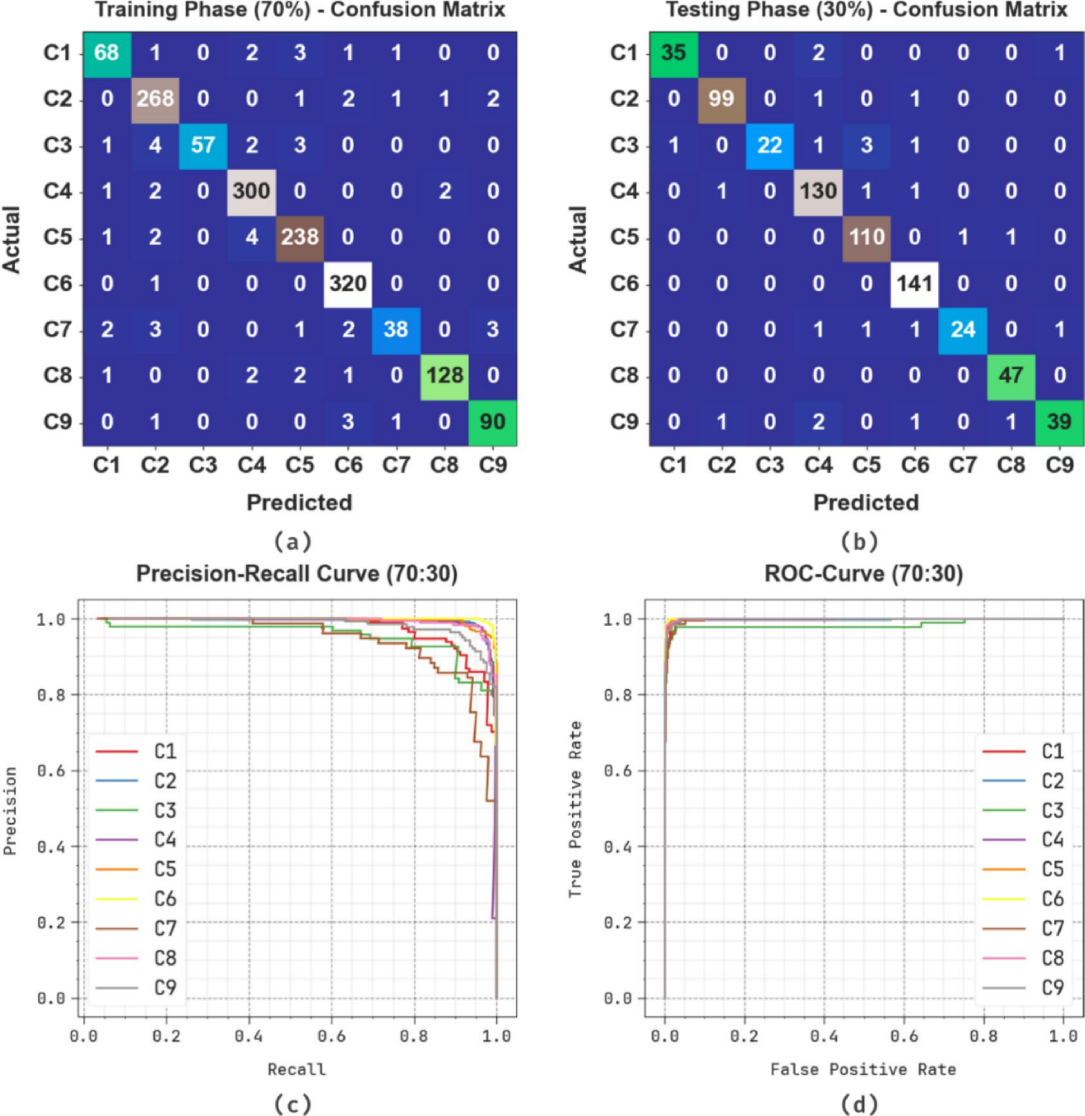
70%TRAPHA and 30%TESPHA of (**a**, **b**) confusion matrices, (**c**) curve of PR, and (**d**) curve of ROC.

Figure 14 illustrates the classification performance of the SLCBSBE-FTLHO model under 70%TRAPHA and 30%TESPHA on a multi-class dataset comprising nine classes labelled C1 to C9. In TRAPHA, most classes show high true positive counts, particularly C4, C5, and C6, indicating effective learning and minimal misclassifications. The TESPHA maintains this trend with notable accuracy for classes C2, C4, C5, C6, and C9, highlighting robust diagonal dominance. Although minor misclassifications are present in classes such as C3 and C7, they are relatively limited and do not significantly affect the overall performance. The model depicts consistent generalization capability, a good balance between precision and recall, and promising classification stability across the dataset.

**Fig. 15:**
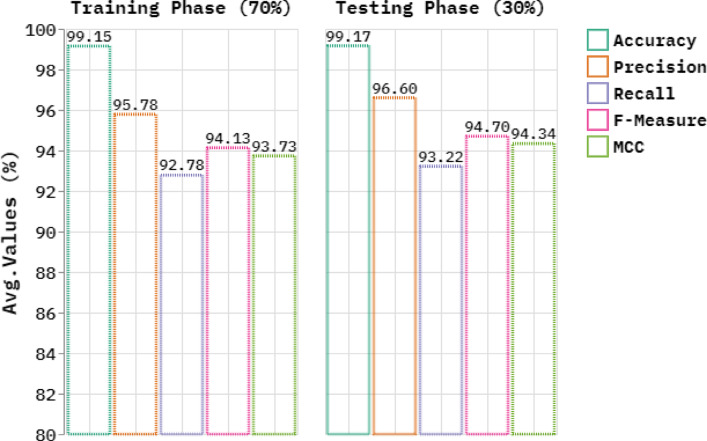
Average of SLCBSBE-FTLHO model under 70%TRAPHA and 30%TESPHA.

Table 3; Fig. 15 represent the skin cancer detection by the SLCBSBE-FTLHO method under 70%TRAPHA and 30%TESPHA. The table values indicate that the SLCBSBE-FTLHO model correctly recognized the samples. By 70%TRAPHA, the SLCBSBE-FTLHO model bids average $\:acc{u}_{y}$, $\:pre{c}_{n}$, $\:rec{a}_{l},\:{F}_{Measure}$, and $\:MCC$ of 99.15%, 95.78%, 92.78%, 94.13%, and 93.73%, correspondingly. Likewise, with 30%TESPHA, the SLCBSBE-FTLHO method offers average $\:acc{u}_{y}$, $\:pre{c}_{n}$, $\:rec{a}_{l},\:{F}_{Measure}$, and $\:MCC$ of 99.17%, 96.60%, 93.22%, 94.70%, and 94.34%, respectively.

**Table 3:** Skin cancer detection of SLCBSBE-FTLHO model under 70%TRAPHA and 30%TESPHA.

Classes	$\:\varvec{A}\varvec{c}\varvec{c}{\varvec{u}}_{\varvec{y}}$	$\:\varvec{P}\varvec{r}\varvec{e}{\varvec{c}}_{\varvec{n}}$	$\:\varvec{R}\varvec{e}\varvec{c}{\varvec{a}}_{\varvec{l}}$	$\:{\varvec{F}}_{\varvec{m}\varvec{e}\varvec{a}\varvec{s}\varvec{u}\varvec{r}\varvec{e}}$	$\:\varvec{M}\varvec{C}\varvec{C}$
**TRAPHA (70%)**					
C1	99.11	91.89	89.47	90.67	90.21
C2	98.66	95.04	97.45	96.23	95.43
C3	99.36	100.00	85.07	91.94	91.93
C4	99.04	96.77	98.36	97.56	96.97
C5	98.92	95.97	97.14	96.55	95.91
C6	99.36	97.26	99.69	98.46	98.07
C7	99.11	92.68	77.55	84.44	84.34
C8	99.43	97.71	95.52	96.60	96.30
C9	99.36	94.74	94.74	94.74	94.40
**Average**	**99.15**	**95.78**	**92.78**	**94.13**	**93.73**
**TESPHA (30%)**					
C1	99.40	97.22	92.11	94.59	94.32
C2	99.40	98.02	98.02	98.02	97.67
C3	99.11	100.00	78.57	88.00	88.23
C4	98.51	94.89	97.74	96.30	95.38
C5	98.96	95.65	98.21	96.92	96.30
C6	99.26	96.58	100.00	98.26	97.81
C7	99.26	96.00	85.71	90.57	90.34
C8	99.70	95.92	100.00	97.92	97.78
C9	98.96	95.12	88.64	91.76	91.27
**Average**	**99.17**	**96.60**	**93.22**	**94.70**	**94.34**

**Fig. 16:**
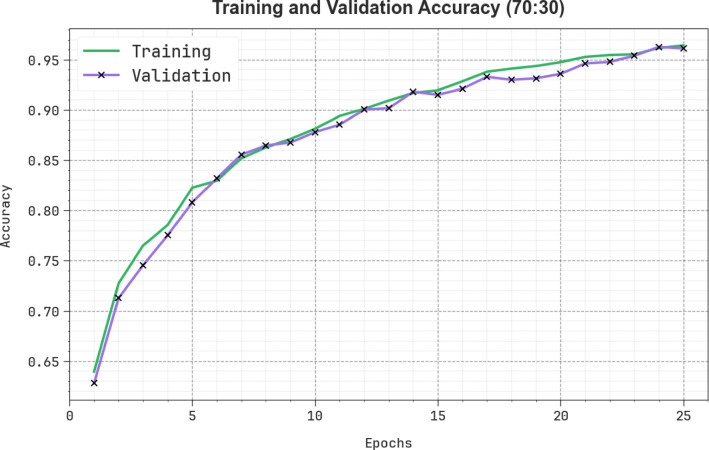
$\:Acc{u}_{y}$ curve of SLCBSBE-FTLHO model under 70:30

Figure 16 shows the TRAN $\:acc{u}_{y}$ and VALN $\:acc{u}_{y}$ results of the SLCBSBE-FTLHO technique under 70:30. The $\:acc{u}_{y}\:$values are estimated through an interval of 0–25 epochs. The figure emphasized that both $\:acc{u}_{y}$ Values exhibit a growing tendency, which indicates the capacity of the SLCBSBE-FTLHO technique with improved outcomes over some iterations. Additionally, both $\:acc{u}_{y}$ stays closer on the epochs, which states the least overfitting and reveals boosted performance of the SLCBSBE-FTLHO model, ensuring consistent prediction on unseen samples.

**Fig. 17:**
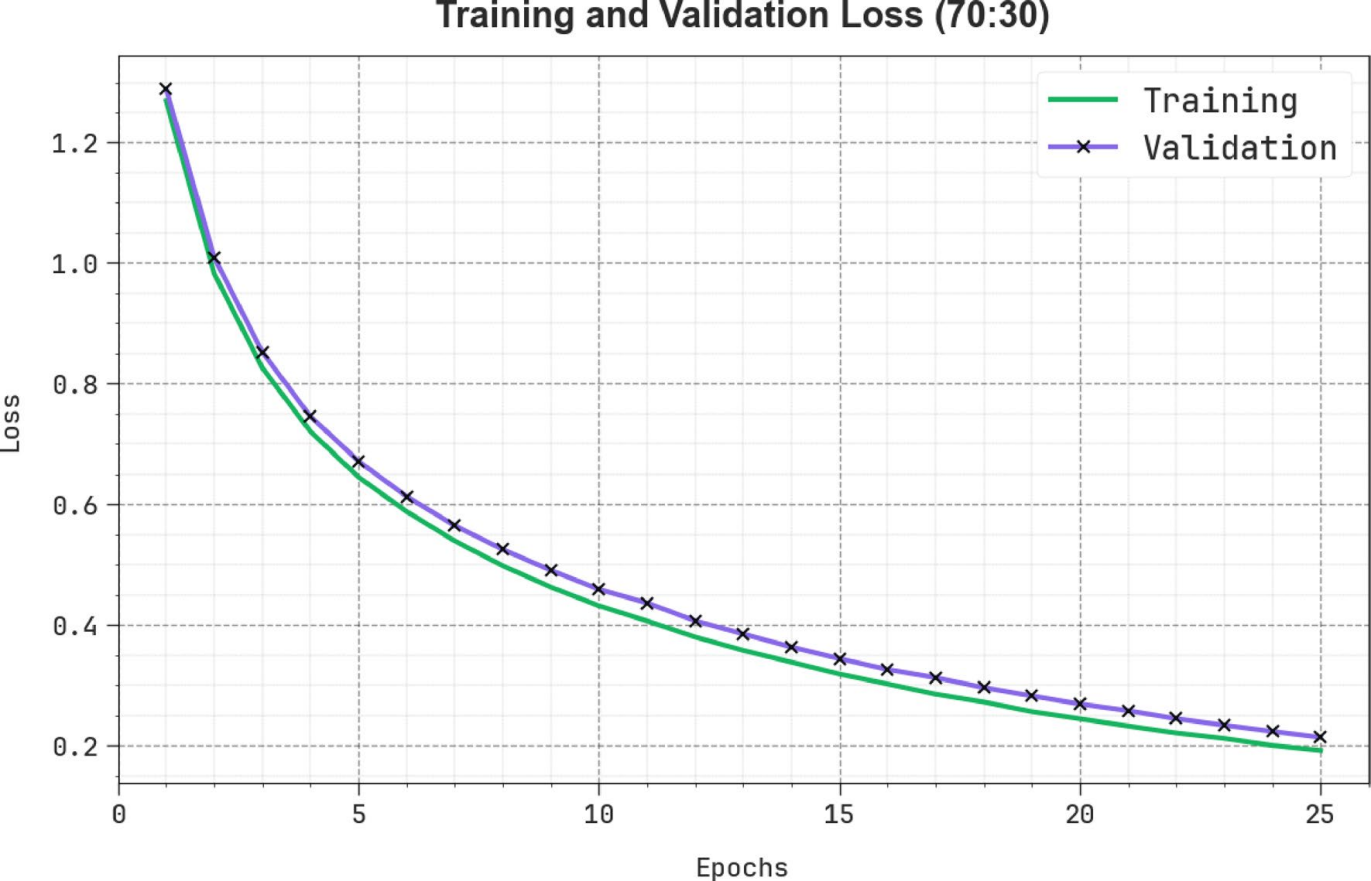
Loss curve of SLCBSBE-FTLHO method under 70:30.

Figure 17 portrays the TRANLOS and VALNLOS chart of the SLCBSBE-FTLHO method under 70:30. The loss values are computed over 0–25 epochs. Both values exemplify a declining leaning that indicates the competence of the SLCBSBE-FTLHO technique in equalizing a trade-off. The continuous decrease in loss values guarantees the enhanced performance of the SLCBSBE-FTLHO technique.

**Fig. 18:**
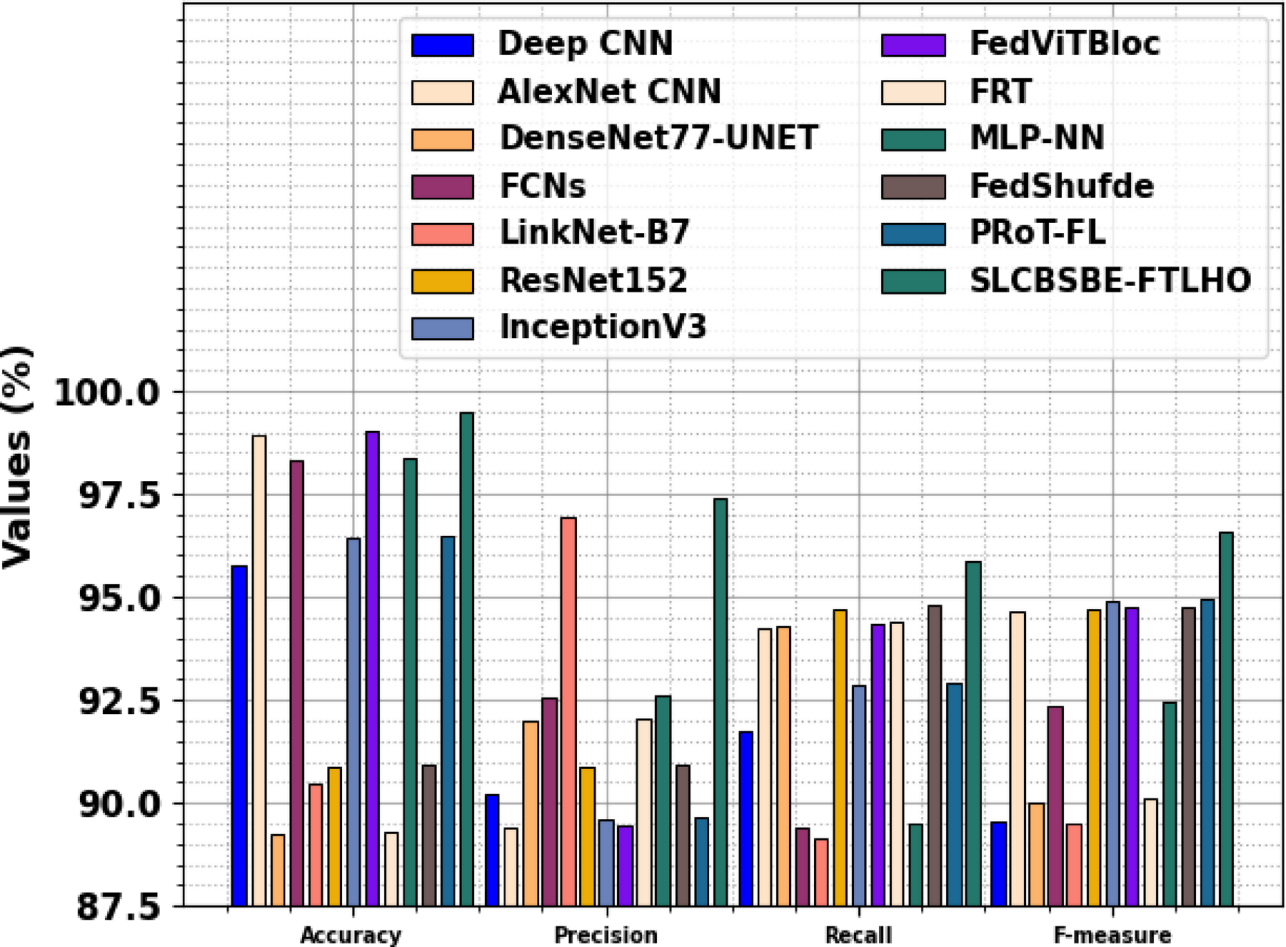
Comparison study of the SLCBSBE-FTLHO approach with existing models.

Table 4; Fig. 18 compare the SLCBSBE-FTLHO structure with recent techniques proven^20–23,39–41^. The findings underlined that the current methods like Deep CNN, AlexNet CNN, DenseNet77-UNET, FCNs, LinkNet-B7, ResNet152, InceptionV3, FedViTBloc, FRT, MLP-NN, FedShufde, and PRoT-FL exhibited worst performance. But, the presented SLCBSBE-FTLHO technique stated upgraded performance by maximum $\:acc{u}_{y}$ of 99.48%, $\:pre{c}_{n}$ of 97.40%, $\:rec{a}_{l}$ of 95.86%, and $\:{F}_{Measure}$ of 96.59%.

**Table 4:** Comparison study of the SLCBSBE-FTLHO approach with existing models^20–23,39–41^.

Approach	$\:\varvec{A}\varvec{c}\varvec{c}{\varvec{u}}_{\varvec{y}}$	$\:\varvec{P}\varvec{r}\varvec{e}{\varvec{c}}_{\varvec{n}}$	$\:\varvec{R}\varvec{e}\varvec{c}{\varvec{a}}_{\varvec{l}}$	$\:{\varvec{F}}_{\varvec{m}\varvec{e}\varvec{a}\varvec{s}\varvec{u}\varvec{r}\varvec{e}}$
Deep CNN	95.77	90.21	91.73	89.55
AlexNet CNN	98.95	89.38	94.24	94.67
DenseNet77-UNET	89.23	91.98	94.31	90.01
FCNs	98.30	92.56	89.42	92.38
LinkNet-B7	90.47	96.94	89.13	89.52
ResNet152	90.89	90.87	94.72	94.69
InceptionV3	96.41	89.60	92.84	94.88
FedViTBloc	99.01	89.45	94.32	94.73
FRT	89.29	92.04	94.38	90.09
MLP-NN	98.36	92.62	89.48	92.45
FedShufde	90.96	90.95	94.80	94.75
PRoT-FL	96.48	89.68	92.91	94.96
SLCBSBE-FTLHO	99.48	97.40	95.86	96.59

**Fig. 19:**
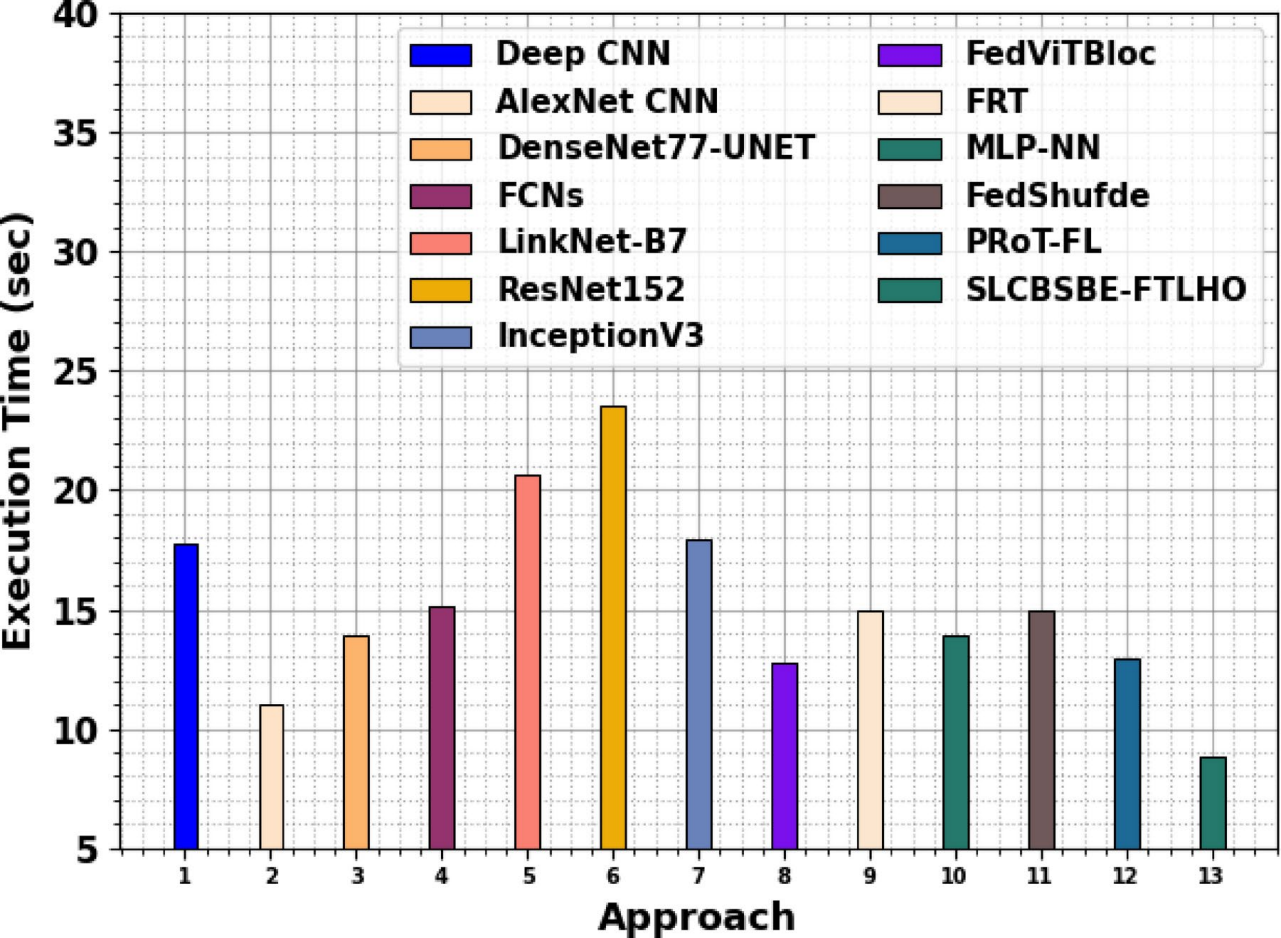
ET outcome of SLCBSBE-FTLHO methodology with existing methods.

In Table 5; Fig. 19, the execution time (ET) result of the SLCBSBE-FTLHO method with recent models is presented. The introduced SLCBSBE-FTLHO technique presents less ET of 8.85 s, while the Deep CNN, AlexNet CNN, DenseNet77-UNET, FCNs, LinkNet-B7, ResNet152, InceptionV3, FedViTBloc, FRT, MLP-NN, FedShufde, and PRoT-FL procedures accomplish larger ET of 17.75 s, 11.02 s, 13.95 s, 15.10 s, 20.61 s, 23.52 s, 17.92 s, 12.78 s, 15.00 s, 13.91 s, 15.00 s, and 12.90 s, correspondingly.

**Table 5:** ET outcome of SLCBSBE-FTLHO methodology with existing methods^20–23,39–41^.

Approach	ET (sec)
Deep CNN	17.75
AlexNet CNN	11.02
DenseNet77-UNET	13.95
FCNs	15.10
LinkNet-B7	20.61
ResNet152	23.52
InceptionV3	17.92
FedViTBloc	12.78
FRT	15.00
MLP-NN	13.91
FedShufde	15.00
PRoT-FL	12.90
SLCBSBE-FTLHO	8.85

**Fig. 20:**
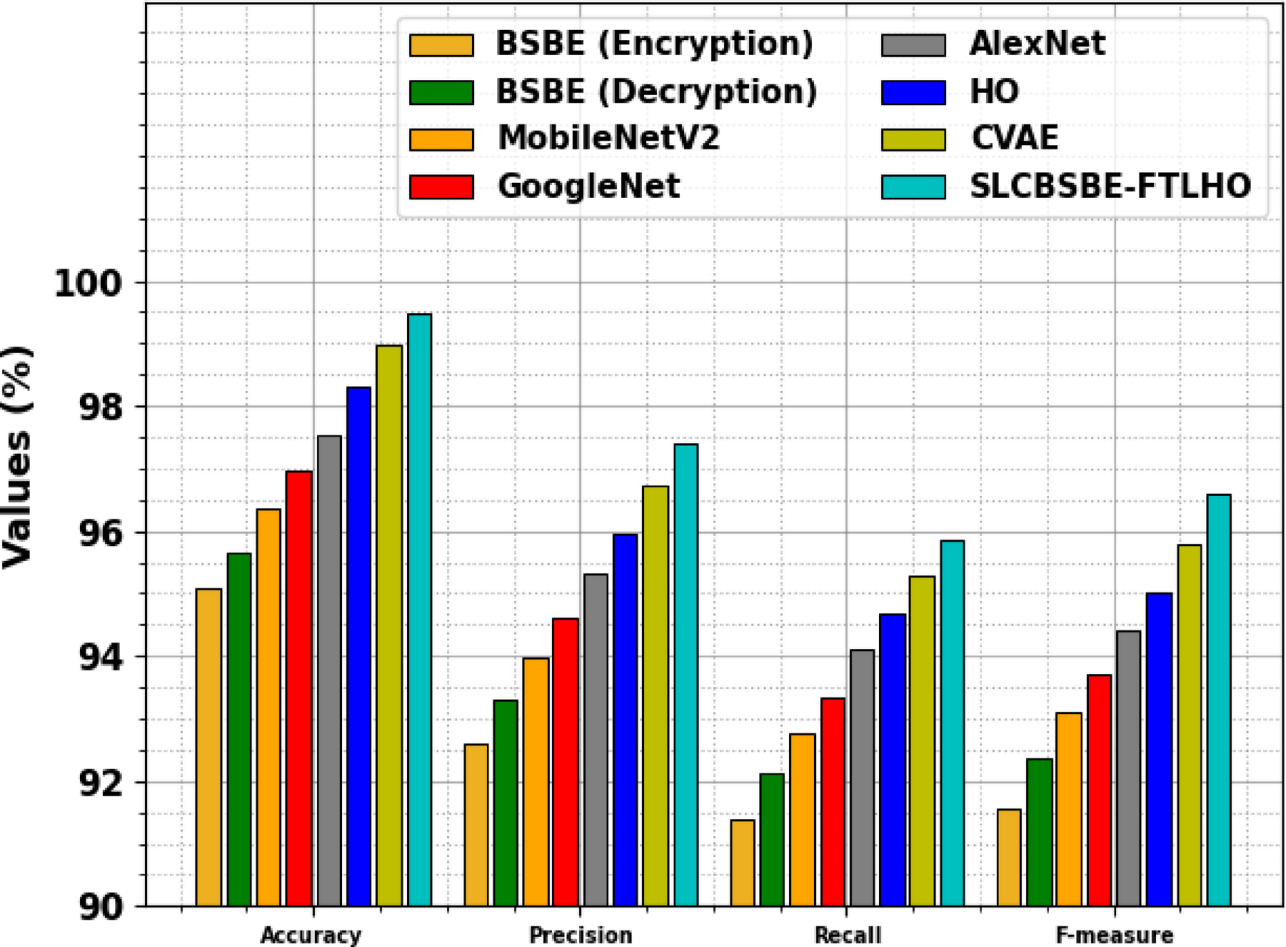
Result analysis of the ablation study of SLCBSBE-FTLHO approach.

Table 6; Fig. 20 demonstrate the ablation study of the SLCBSBE-FTLHO approach. The BSBE encryption and decryption stages achieved $\:acc{u}_{y}$ of 95.09% and 95.66% with $\:{F}_{Measure}$ of 91.55% and 92.35%, respectively. Furthermore, MobileNetV2 reached an $\:acc{u}_{y}$ of 96.35% and $\:{F}_{Measure}$ of 93.11%, while AlexNet exhibited improved $\:acc{u}_{y}$ of 97.55% and 94.42% F1-score. The HO illustrated an $\:acc{u}_{y}$ of 98.29% and $\:{F}_{Measure}$ of 95.03%. With the addition of the CVAE, an $\:acc{u}_{y}$ of 98.98% and $\:{F}_{Measure}$ of 95.79% is attained. Finally, the SLCBSBE-FTLHO model achieved the highest $\:acc{u}_{y}$ of 99.48% and $\:{F}_{Measure}$ of 96.59%, highlighting the superiority of the integrated approach in improving classification performance. This analysis emphasizes the individual impact of each component, illustrating how their combination significantly improves overall performance.

**Table 6:** Result analysis of the ablation study of SLCBSBE-FTLHO approach.

Approach	$\:\varvec{A}\varvec{c}\varvec{c}{\varvec{u}}_{\varvec{y}}$	$\:\varvec{P}\varvec{r}\varvec{e}{\varvec{c}}_{\varvec{n}}$	$\:\varvec{R}\varvec{e}\varvec{c}{\varvec{a}}_{\varvec{l}}$	$\:{\varvec{F}}_{\varvec{m}\varvec{e}\varvec{a}\varvec{s}\varvec{u}\varvec{r}\varvec{e}}$
BSBE (encryption)	95.09	92.61	91.39	91.55
BSBE (decryption)	95.66	93.31	92.14	92.35
MobileNetV2	96.35	93.99	92.78	93.11
GoogleNet	96.96	94.61	93.34	93.70
AlexNet	97.55	95.33	94.12	94.42
HO	98.29	95.94	94.67	95.03
CVAE	98.98	96.72	95.28	95.79
SLCBSBE-FTLHO	99.48	97.40	95.86	96.59

## Conclusion

This paper presents an SLCBSBE-FTLHO model. The main goal of the SLCBSBE-FTLHO model relies on automating the diagnostic procedures of skin lesions utilizing optimal DL models. At first, the BSBE technique is used in the image encryption pre-processing stage, and then the decryption process is performed. The feature extraction process employs the fusion of MobileNetV2, GoogLeNet, and AlexNet techniques. Furthermore, the CVAE method employs the SLCBSBE-FTLHO model for the skin lesion classification. To further optimize the CVAE model performance, the HO model is utilized for hyperparameter tuning to ensure that the optimum hyperparameters are chosen for enhanced accuracy. To exhibit the improved performance of the SLCBSBE-FTLHO approach, a comprehensive experimental analysis is conducted under the skin cancer ISIC dataset. The comparative study of the SLCBSBE-FTLHO approach portrayed a superior accuracy value of 99.48% over existing models. The limitations of the SLCBSBE-FTLHO approach include reliance on a fixed dataset, which may not generalize well to various skin tones, lighting conditions, or imaging devices. Furthermore, the model may encounter difficulty in precisely classifying rare or less common skin diseases due to data imbalance. The encryption and decryption stages may present computational overhead, affecting real-time deployment in low-resource settings. The framework does not integrate patient metadata, which may improve the prediction accuracy. Also, there is limited exploration of explainability, which is significant for clinical acceptance. A research gap exists in computing the system’s robustness against adversarial attacks. Future works may explore incorporating massive and more diverse datasets, enhancing computational efficiency, and integrating explainable AI techniques for greater transparency.

## Data Availability

The data supporting this study’s findings are openly available in the Kaggle repository at https://www.kaggle.com/datasets/nodoubttome/skin-cancer9-classesisic, reference number^36^.
